# PSMA1 mediates tumor progression and poor prognosis of gastric carcinoma by deubiquitinating and stabilizing TAZ

**DOI:** 10.1038/s41419-022-05417-0

**Published:** 2022-11-23

**Authors:** Qinyu Yang, Ying Lu, Jianfang Shangguan, Xu Shu

**Affiliations:** Department of Gastroenterology, Digestive Disease Hospital, The First Affiliated Hospital of Nanchang University, Nanchang, China

**Keywords:** Tumour biomarkers, Clinical genetics

## Abstract

The deubiquitinating enzyme family in tumor progression play important role in intracellular protein degradation. The proteasome subunit alpha type 1 (PSMA1) has been reported to act as an oncogene in several human cancers. The present study aimed to reveal the functional significance of PSMA1 in gastric cancer (GC) progression and the underlying mechanisms. The expression of PSMA1 in human GC samples and GC cell lines was examined by western blot analysis, real-time PCR, immunohistochemistry (IHC), and in vitro ubiquitination assays and established a xenograft mouse model. We found that PSMA1 was upregulated in GC and promoted proliferation, migration and invasion in GC cells. Herein, we report transcriptional co-activator with PDZ-binding motif (TAZ) was a downstream gene of PSMA1. Mechanistically, PSMA1 directly interacted with and stabilized TAZ via deubiquitination in GC. Furthermore, we found that TAZ was the essential mediator of PSMA1-modulated oncogenic activity in vitro and in vivo. Examination of clinical samples confirmed that elevated mediators of PSMA1, concomitant with increased TAZ abundance, correlate with human GC progression. These data suggested that PSMA1 promotes GC progression and proliferation by deubiquitinating TAZ. PSMA1 promotes GC progression and proliferation regarding PSMA1-mediated deubiquitinating enzyme activity and suggest potential therapeutic targets for GC management.

## Introduction

Gastric cancer (GC) was the fifth most common malignancy and the fourth leading cause of cancer-related deaths worldwide [1]. Although the incidence of GC is decreasing at an annual rate of about 2% per year, the number of cases and deaths is expected to increase in the coming years due to the increasing number of aging populations [2]. Due the mechanism of GC progression remains complex to understand, finding new promising molecular markers could provide a new treatment for improving the clinical outcome and prognosis of GC patients.

Ubiquitination is a post-translational modification that plays a critical role in a diverse array of cellular processes [3]. Ubiquitination is one of the most common post-translational modifications. Ubiquitination refers to the process of specific modification of target proteins by ubiquitin under the catalysis of a series of enzymes. It considered to be an important way of protein post-translational modification. It plays an important role in cellular processes such as apoptosis, cell cycle regulation, DNA damage repair, and membrane transport [4]. Like other post-translational modifications, ubiquitination can be reversed by the activation of deubiquitinates. Deubiquitinating enzymes (DUB) can release ubiquitin from the breakdown of substrate proteins, edit ubiquitin chains, process ubiquitin precursors, improve target protein stability, alter cellular localization, or alter protein-protein interactions. Many studies focus on deubiquitinates to discover new therapeutic targets in tumor therapy [5–7]. The 26S proteasome is a large multi-subunit protein complex consisting of a 20S core complex and a 19S regulatory complex. The 20S core complex contains seven distinct alpha (PSMA1-7) and beta (PSMB1-7) subunits [8]. The proteasome subunit alpha type 1 (PSMA1) is a multicatalytic proteinase complex with a highly ordered ring-shaped 20S core structure. Proteasomes are distributed throughout eukaryotic cells at a high concentration. PSMA1 has served as a DUB and is related to the cytotoxic effects of bortezomib in myelodysplastic syndrome/acute myeloid leukemia [9]. Yang et al. [10] found that PSMA1 was significantly upregulated in cancer tissues by extracting proteins from colon cancer patients’ cancer tissues and their paired normal tissues, which may be a marker for colon cancer screening and early diagnosis. Results from other studies show overexpression of PSMA1 in the serum of metastatic gastric cancer [11]. However, there are no reports on the details of PSMA1 act as an essential oncogene in GC.

To further explore the role of PSMA1 and its downstream mechanisms, we performed a mass spectrometry analysis. Herein, we reported TAZ as a critical target of PSMA1. The Hippo signaling effector proteins yes-associated protein (YAP)/TAZ have been proved associated with the progression of GC [12]. Both TAZ and its homologous protein YAP are key effector molecules in the Hippo signaling pathway, regulating cell proliferation and apoptosis [13]. Overexpressed YAP and TAZ coordinately enhance the activation of TEAD-dependent transcription of the cell proliferation gene CTGF, induce cancer stem-like properties, and promote tumor cell proliferation [14]. Inhibiting the abnormal expression of YAP/TAZ can delay tumor progression. When YAP and TAZ are deregulated, amplified, or fused into genes encoding transcription factors, they act as potent oncogenes [15]. The previous study mainly focused on individual components of the Hippo signal pathway but how the external pathway affected YAP/TAZ is still unknown. It has been reported that TAZ protein stability is ubiquitylated by the SCF/CRL1(β-TrCP) E3 ligase [16]. Since there is no known therapy target TAZ at the protein level, the therapeutic intervention points in TAZ-driven malignancies outside of the Hippo signal pathway need to be determined.

Recently, several studies had indicated the relation between DUB and TAZ in various cancer [17, 18]. However, there are currently no known DUBs that regulate TAZ specifically in GC. Therefore, we analyzed the specific mechanism of PSMA1-mediated deubiquitination of TAZ. Moreover, we analyzed the effect of such a post-translational regulation axis on progression and proliferation in GC.

In this study, we found PSMA1 high expression in GC tissue compared to normal tissue. Overexpression of PSMA1 significantly promoted proliferation, migration, and invasion both in vitro and in vivo. In addition, we identified PSMA1 as a novel deubiquitinating mediator that stabilizes TAZ in GC. Our results showed that PSMA1 may serve as a therapeutic target for the pathogenesis of GC.

### Statistical analysis

The data were statistically analyzed with the SPSS software package (SPSS, IL, USA) and GraphPad Prism software (GraphPad, CA, USA). Quantitative data obtained from experiments with biological replicates are shown as mean ± standard deviation. Differences between the two groups were assessed by Student’s *t* test, and differences between multiple groups were assessed by one-way ANOVA. Pearson correlation coefficients were used to evaluate the correlation. Survival curves were used the Kaplan–Meier (K–M) method and compared using the log-rank test. *P* < 0.05 was considered statistically significant.

## Results

### The expression of PSMA1 correlates with the progression of GC

**Fig. 1:**
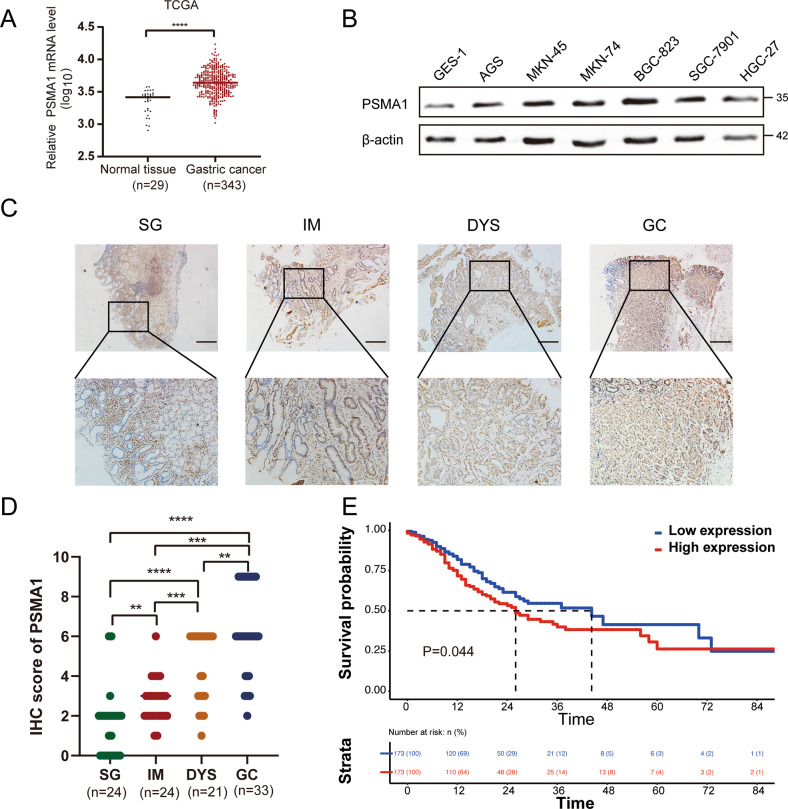
PSMA1 was elevated in gastric cancer. **A** The real-time PCR assay of the mRNA expression of PSMA1 in gastric cancer tissue and normal tissue is based on the data from the TCGA database. **B** The basic PSMA1 protein level in different gastric cancer (GC) cell types. **C** Immunohistochemistry (IHC) staining for PSMA1 in superficial gastritis (SG), intestinal metaplasia (IM), dysplasia (DYS), and GC samples. Bar length: 100 μm. **D** IHC score analyzed the expression levels of PSMA1 in SG, IM, DYS, and GC. **E** The Kaplan–Meier plot of overall survival by the expression of PSMA1 in the GC patients, the data was carried out from the TCGA database.

To explore the clinical function of PSMA1 in human GC, we used RNA-seq from The Cancer Genome Atlas (TCGA) to analyze the mRNA expression of PSMA1 in GC. The results showed that the expression level of PSMA1 in tumor tissues was upregulated compared to adjacent normal tissues (Fig. 1A). Next, we measured the PSMA1 expression level in different GC cell lines and found higher PSMA1 protein levels in GC cells compared to low PSMA1 protein levels in GES-1 cells (Fig. 1B). Immunohistochemistry (IHC) staining showed that PSMA1 expression was relatively low in superficial gastritis (SG), upregulated in intestinal metaplasia (IM), moderately increased in dysplasia (DYS), and strikingly upregulated in GC tissues (Fig. 1C, D). The K-M plot of overall survival by the expression of PSMA1 in the GC patients showed high-level expression of PSMA1 significantly correlated with the poor overall survival of GC patients (Fig. 1E).

### PSMA1 interacts with and deubiquitinates TAZ

**Fig. 2:**
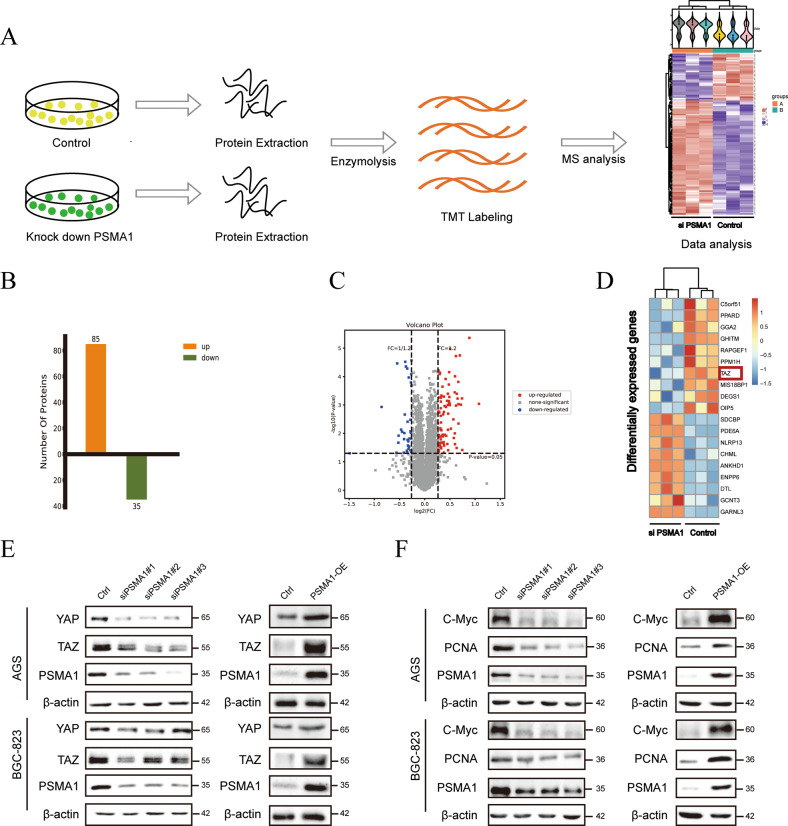
PSMA1 maintains TAZ stability. **A** Framework of the TMT labeling quantitative proteomic study**. B** Upregulated and down-regulated protein by mass spectroscopic analysis. **C** Volcano plot showing differentially expressed proteins. **D** Heatmap shows a differentially expressed gene. **E** Western blot detected YAP and TAZ protein in AGS and BGC-823 cells after transfecting cells with three independent siRNA. Western blot detected YAP and TAZ protein in AGS and BGC-823 cells after transfecting PSMA1-overexpression plasmid. **F** Western blot detected C-Myc and PCNA protein in AGS and BGC-823 cells after transfecting cells with three independent siRNA or PSMA1-overexpression plasmid.

**Fig. 3:**
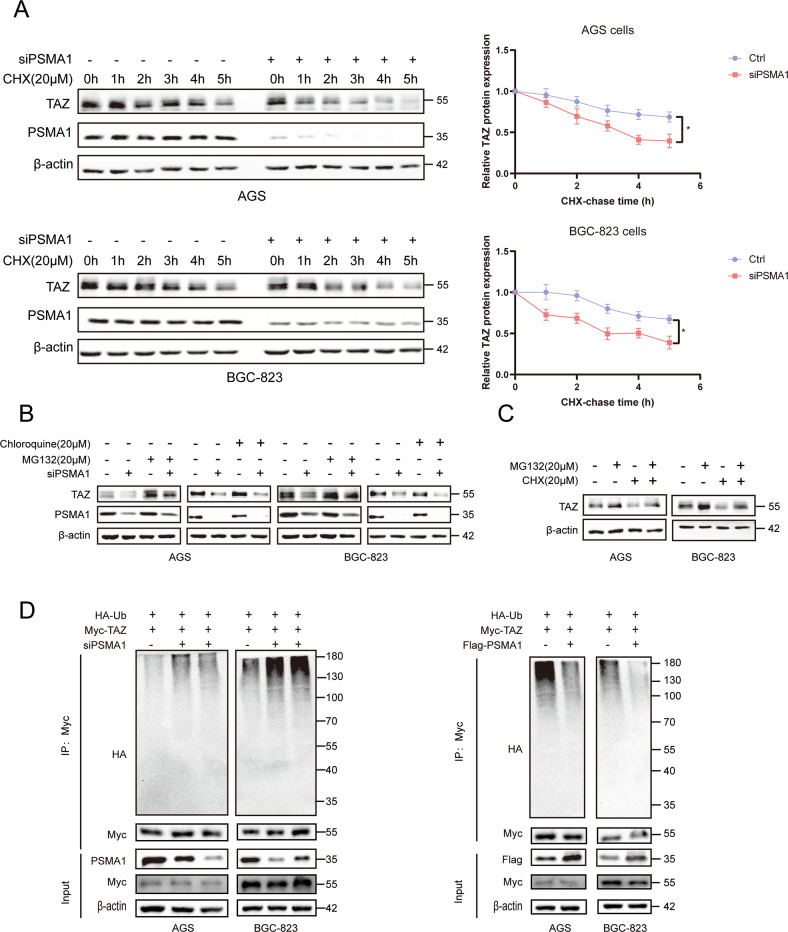
PSMA1 stabilized TAZ through deubiquitination. **A** AGS and BGC-823 cells transfected with siPSMA1 were treated with 20 μM cycloheximide (CHX) for 0, 1, 2, 3, 4, and 5 h, and the PSMA1 and TAZ protein levels were detected by western blot. **B** Western blotting was conducted to measure TAZ protein level after treatment with 20 μM MG132 and treatment with 20 μM Chloroquine (CQ) in knock-down PSMA1 and control GC cells. **C** AGS and BGC-823 cells were treated with 20 μM MG132 accompanied by 20 μM CHX for 6 h. **D** Western blotting was conducted to measure the level of ubiquitin when knocking down or overexpression PSMA1.

To detect the specific substrate proteins that may serve as potential interaction targets of PSMA1, we performed a TMT labeling experiment to systematically detect the expression changes in the protein profile of the PSMA1-Knockdown BGC-823 cell, compare with the control (Fig. 2A). There are 34 downregulated and 86 upregulated proteins identified with significant expression alterations (Fig. 2B, C). Heatmap showing differentially expressed gene, TAZ was significantly downregulated when PSMA1 was knocked down in the TMT experiment (Fig. 2D). As expected, PSMA1 siRNA substantially reduced YAP and TAZ protein levels. Consistently, the overexpression of PSMA1 in AGS and BGC-823 cells greatly upregulated the protein level of YAP/TAZ (Fig. 2E). In addition, knock down PSMA1 reduced proliferation indicators PCNA and C-Myc expression, overexpression PSMA1 upregulated PCNA and C-Myc expression (Fig. 2F). However, real-time quantitative reverse transcription (qRT-PCR) analysis showed that neither knockdown nor overexpression of PSMA1 influenced the mRNA levels of TAZ, which demonstrated that PSMA1 upregulated the expression of TAZ at the post-transcriptional level (Supplementary Fig. 1A, B). We further verified whether PSMA1 impacted the stability of TAZ. A cycloheximide (CHX) chase assay showed that transfected with PSMA1 siRNA accelerated the degradation of TAZ protein in AGS and BGC-823 (Fig. 3A). To determine whether the reduction of the TAZ protein levels in siPSMA1 occurs through proteasome degradation, we treated the siCtrl or siPSMA1 GC cells with the proteasome inhibitor MG132 or autophagy-lysosome inhibitor Chloroquine (CQ). Accordingly, we found that the proteasome inhibitor treatment rather than autophagy-lysosome inhibitor significantly rescued the TAZ protein levels in the absence of PSMA1, indicating that PSMA1 stabilized TAZ and protects it from proteasome degradation (Fig. 3B). Furthermore, we treated GC cells with CHX to inhibit protein synthesis, assay of both cell lines showed that this process could be inhibited by MG132 (Fig. 3C). In addition, the knockdown of PSMA1 dramatically increased the poly-ubiquitination of TAZ. In contrast, the overexpression of PSMA1 gets the opposite result (Fig. 3D). Taken together, these results indicated that PSMA1 is a potent deubiquitinating enzyme TAZ for deubiquitination and stabilization.

### PSMA1 promoted the proliferation, migration, and invasion of GC cell lines

**Fig. 4:**
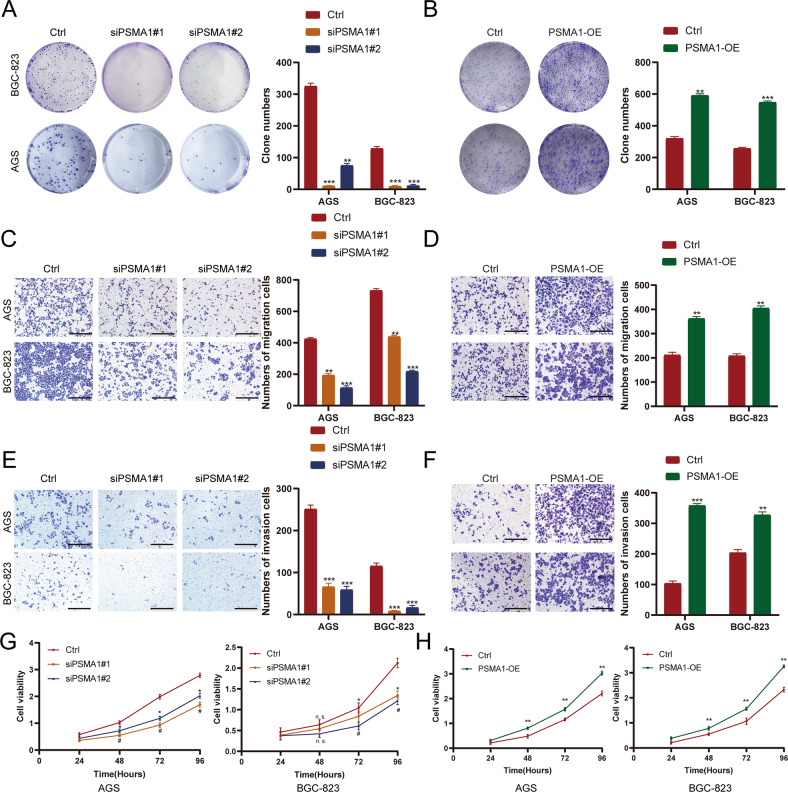
PSMA1 promoted the proliferation, migration, and invasion of GC cell lines. **A** Colony formation assay was performed to detect the proliferation of knock-down PSMA1 and control GC cells. **B** Colony formation assay was performed to detect the proliferation of over-expression PSMA1 and control GC cells. **C**, **E** Invasion and migration assay were performed to detect the proliferation of knock down PSMA1 and control GC cells. Bar length: 100 μm. **D**, **F** Invasion and migration assays were performed to detect the proliferation of over-expression PSMA1 and control GC cells. Bar length: 100 μm. **G** The CCK-8 assay was used to detect the proliferation of knock-down PSMA1 and control GC cells. **H** The CCK-8 assay was used to detect the proliferation of over-expression PSMA1 and control GC cells.

To investigate the potential function of PSMA1 in GC progression, we knockdown and overexpression PSMA1 in AGS and BGC-823 cells using siPSMA1 and overexpression plasmid. The colony assay showed that the colony numbers were significantly reduced in PSMA1 knockdown GC cells compared with control group cells (Fig. 4A). We performed a transwell migration assay to further evaluate the impact of PSMA1 on cell migration. The results indicated that knockdown of PSMA1 significantly reduced the migration of GC cells, and overexpression of PSMA1 significantly facilitated the migration of GC cells (Fig. 4C, D). Considering the important role of invasion in the development of GC, a transwell assay was executed to evaluate the influence of PSMA1-knockdown and PSMA1-overexpression on invasion. The results showed that PSMA1 upregulated and promoted the invasion of GC cells (Fig. 4E, F). The Cell Counting Kit-8 (CCK-8) assay showed that PSMA1 suppression decreased the viability of GC cells (Fig. 4G). PSMA1 overexpression significantly promoted GC cell proliferation and colony formation, as determined by CCK-8 and colony formation assays, respectively (Fig. 4H, B). These results indicate that PSMA1 facilitated the growth and proliferation of GC cells, suggesting a tumor promotor role for PSMA1 in GC.

### PSMA1 exerts oncogenic effects through TAZ

**Fig. 5:**
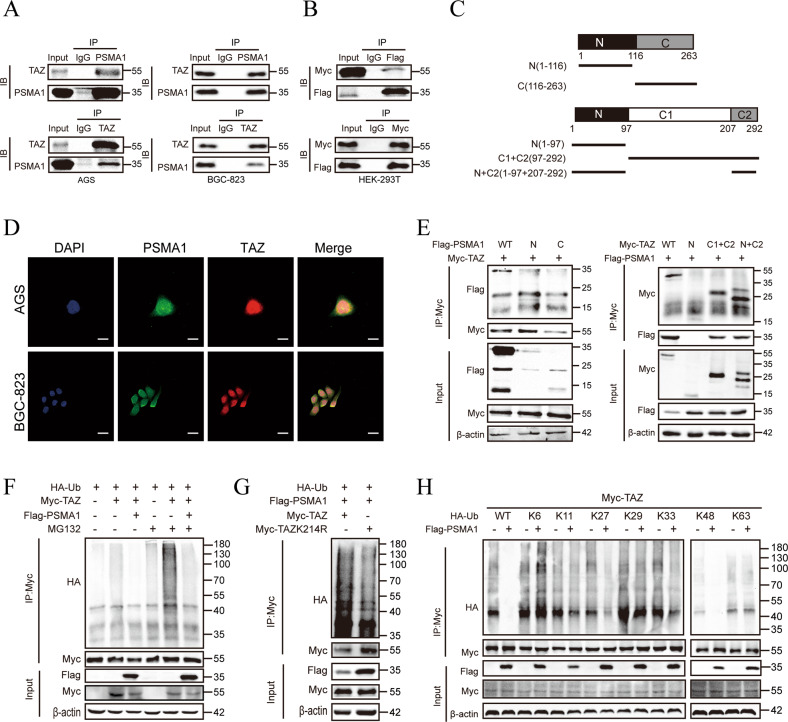
PSMA1 interacting with TAZ via deubiquitination. **A** Endogenous PSMA1 proteins were immunoprecipitated with an anti-PSMA1 antibody and then analyzed by immunoblotting. Endogenous TAZ proteins were immunoprecipitated with anti-TAZ antibodies and then analyzed by immunoblotting. The IgG antibody was used as the control **B** HEK-293T cells co-transfected with Flag-PSMA1 and Myc-TAZ plasmid were subject to immunoprecipitation with anti-Flag and anti-Myc antibodies. **C** Segment plasmid of PSMA1 and TAZ. **D** Immunofluorescence assays for PSMA1 and TAZ in GC cells were performed to detect co-location. Bar length: 20 μm. **E** HEK-293T cells were transfected with Myc-TAZ and Flag-PSMA1 or several PSMA1 deletion mutants. HEK-293T cells were transfected with Flag-PSMA1 and Myc-TAZ or several TAZ deletion mutants. A coimmunoprecipitation assay was performed to detect the interaction between PSMA1 and TAZ protein. **F** HEK-293T cells were co-transfected with HA-Ub, Myc-TAZ, and Flag-PSMA1 plasmid. After 48 h, the cells were treated with 20 μM MG132 for 6 h. Representative WB analyses of ubiquitinated TAZ with or without PSMA1 overexpression. **G** Representative WB analysis showing the influence of mutation of TAZ on PSMA1 ubiquitin. **H** HEK-293T cells were co-transfected HA-WT, K6, K11, K27, K29, K33, K48, or K63 Ub with Myc-TAZ and Flag-PSMA1 plasmid. Cell lysates were subjected to ubiquitination assay and the ubiquitination level of TAZ was detected by HA antibody.

To illustrate the mechanisms responsible for the effects of PSMA1 in maintaining the stability of TAZ, we performed reciprocal co-immunoprecipitation (Co-IP) experiments using endogenous protein. The results showed that PSMA1 could interact with TAZ (Fig. 5A). Furthermore, exogenous Flag-tagged PSMA1 and Myc-tagged TAZ plasmid were co-transfected into HEK-293T cells, we found that Flag-tagged PSMA1 co-immunoprecipitated with Myc-tagged TAZ (Fig. 5B). Additionally, we performed an immunofluorescence assay to identify PSMA1 localization. We found that PSMA1 and TAZ were observed with marked colocalization in AGS and BGC-823 cells (Fig. 5D). The above results strongly support the conclusion that PSMA1 interacts with TAZ in GC cells. To investigate the exact regions where two proteins interact, we constructed a segment plasmid of PSMA1 and TAZ. Co-IP analyses revealed that the N domain of TAZ showed no combined effects with PSMA1 (Fig. 5C, E). The deubiquitination effect of PSMA1 on TAZ was examined in subsequent experiments. We transfected the ubiquitin-overexpressed plasmid into HEK-293T cells with or without PSMA1 overexpression and detected the ubiquitination levels of TAZ. Western blotting showed that the ubiquitination of TAZ was strongly inhibited by PSMA1 overexpression. Furthermore, these findings were also observed consistently in the presence or absence of MG132, a potent inhibitor of the 26 S proteasome, which catalyzed the degradation of protein after ubiquitination modification (Fig. 5F). By mass spectrometry, we found TAZ was ubiquitinated on Lysine 214 (Supplementary Fig. 2). We then co-transfected HA-Ub and TAZ-WT or TAZ-K214R in HEK-293T cells, co-immunoprecipitation assay demonstrated the decreased ubiquitination of TAZ in the TAZ-K214R mutant compared to TAZ-WT (Fig. 5G). Conjugation of ubiquitin on this site might therefore affect the ability of TAZ to interact with PSMA1. We also performed a ubiquitination essay with a series of mutant ubiquitin. The result showed that PSMA1 could only remove the K27- and K48-linked ubiquitin chain from TAZ protein (Fig. 5H). We further investigated the effect of ubiquitinated on Lysine 214 site of TAZ on its function of promoting proliferation, migration and invasion of AGS cells. The CCK-8 and colony formation assays proved that the promoting effect of TAZ on cell activity and proliferation was abolished by mutation of Lysine 214 to Arginine (K214R) (Supplementary Fig. 3A, B). Moreover, the regulation of TAZ on migration and invasion were also abrogated by K214R site (Supplementary Fig. 3C). In conclusion, these results demonstrated that TAZ promotes GC depending on its lysine 214 site. Collectively, these results indicated that PSMA1 maintained the stability of TAZ by decreasing its ubiquitination.

**Fig. 6:**
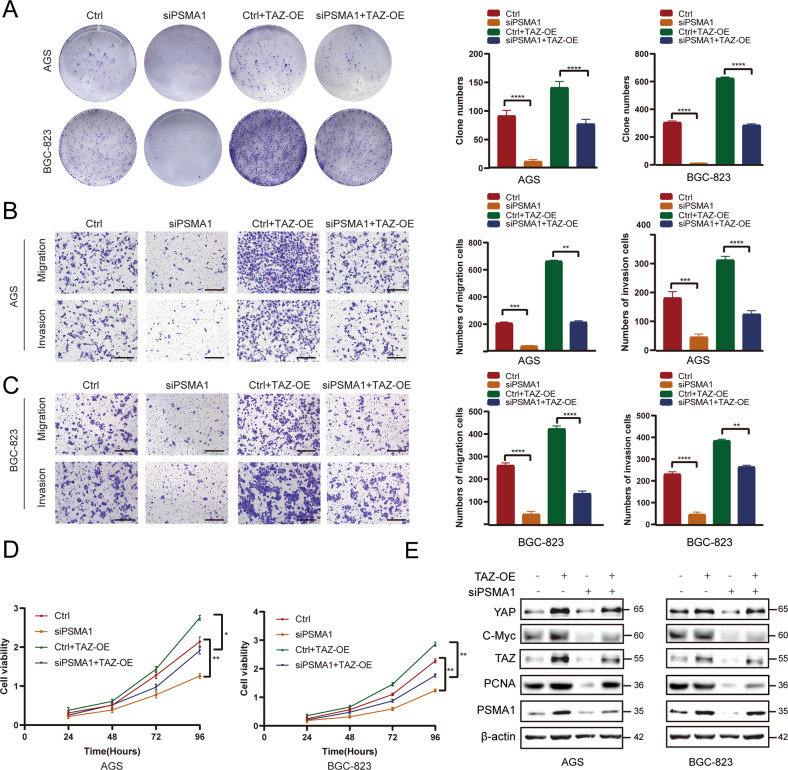
Oncogenic effect of PSMA1 is dependent on TAZ stabilization. **A** Colony formation assay was performed to detect the proliferation of GC cells. **B** Invasion and migration assays were performed to detect the proliferation of AGS cells. Bar length: 100 μm. **C** Invasion and migration assays were performed to detect the proliferation of BGC-823 cells. Bar length: 100 μm. **D** CCK-8 assay was used to detect the proliferation of GC cells. **E** Western blot showing TAZ expression and proteins related to cell growth, such as PCNA and C-Myc in GC cells after a knockdown of PSMA1 and overexpression TAZ or only knockdown PSMA1.

Furthermore, we evaluated the effects of TAZ upregulation caused by PSMA1-mediated deubiquitination of PSMA1-induced GC cell proliferation. We set four groups, including the ctrl, PSMA1-knockdown, ctrl with TAZ-overexpressed, and PSMA1-knockdown with TAZ-overexpressed groups, for subsequent cell experiments. Colony formation assay and CCK-8 assay showed the downregulation of PSMA1 reduced the ability of AGS cells to proliferate, while overexpression of TAZ recovered the proliferate ability of PSMA1-knockdown cells (Fig. 6A, D). We then performed a transwell experiment to detect cell invasion and migration. The results showed that downregulation of PSMA1 reduced the ability of AGS cells to invade and migrate, while overexpression of TAZ recovered the invasion and migration abilities of PSMA1-knockdown cells. Consistent results were observed in BGC-823 cells (Fig. 6B, C). Western blot assays in GC cells showed the depletion of PSMA1 decreased the expression of C-Myc and PCNA, while overexpression of TAZ rescued the proliferation ability in PSMA1-knockdown cells (Fig. 6E).

### PSMA1 is positively correlated with TAZ in clinical GC samples and predicts a poor survival outcome

**Fig. 7:**
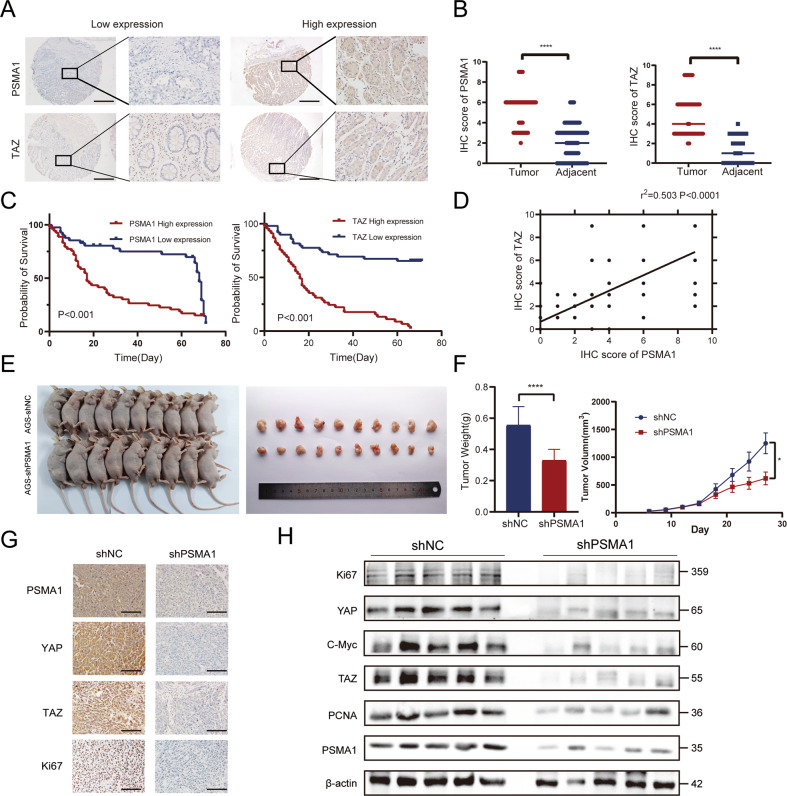
PSMA1 upregulates TAZ to promote GC cell proliferation in vivo. **A** Representative image of IHC staining of tumor microarrays from GC patients. Bar length: 200 μm. **B** IHC scores of PSMA1 and TAZ level between gastric cancer tissue and adjacent normal tissue. **C** The Kaplan–Meier plot of overall survival by the expression of PSMA1 and TAZ in tumor microarrays related information. **D** Pearson’s correlation was used to determine the relationship between PSMA1 and TAZ protein expression in gastric cancer tissue specimens. **E** AGS cells with/without shPSMA1 were injected in nude mice. Representative images of xenograft tumors from mice bearing AGS-shNC and AGS-shPSMA1 cells. **F** Tumor volume was measured every 3 days. Tumor weight was calculated for each mouse. **G** PSMA1, YAP, TAZ, and Ki67 expression in the tumor of nude mice were detected by IHC. Bar length: 100 μm. **H** Western blotting was conducted to measure the level of proliferation-related intratumor protein level.

We analyzed PSMA1 and TAZ expression in tumor samples and adjacent normal tissues from 94 GC patients using tumor microarrays (TMA), along with their corresponding clinicopathologic information. Both PSMA1 and TAZ showed high expression in most tumor specimens (Fig. 7A). IHC scores showed that PSMA1 and TAZ proteins were expressed at significantly higher levels in gastric cancer tissue than that in adjacent normal tissue (Fig. 7B). We collected 20 pairs of GC and SG tissues via endoscopy. Similarly, TAZ showed high expression in tumor tissue (Supplementary Fig. 4A, B). Clinicopathological analysis revealed that high expression of PSMA1 was correlated with late TNM stage (*P* = 0.006) and positive lymph node metastasis (*P* = 0.041; Table 1). We further explored the prognostic effect of such an expression association on survival outcomes of GC patients. The results showed that patients with high expression of PSMA1 had worse prognosis than those with low expression of PSMA1. The same results showed in TAZ protein (Fig. 7C). Moreover, statistically significant correlations were found between PSMA1 and TAZ levels (Fig. 7D).

**Table 1:** Correlation between PSMA1 protein expression and clinicopathologic characteristics of GC.

Variables	Total (*n* = 94)	PSMA1 expression level		*P* value
		High (*n* = 52)	Low (*n* = 42)	
Age				
≤60	27	13	14	0.784
>60	67	39	28	–
Sex				
Male	59	32	27	0.375
Female	35	20	15	–
Tumor type				
Protuberant	7	3	4	0.377
Infiltrative	24	11	13	–
Ulcerative	63	38	25	–
Tumor grade				0.189
I–II	40	19	21	–
III	54	33	21	–
TNM staging				0.006
I/II	37	14	23	–
III/IV	57	38	19	–
Tumor size				
≤5 cm	47	24	23	0.407
>5 cm	47	28	19	–
Lymphovascular invasion				0.049
Yes	25	20	5	–
No	69	32	37	–
Nerve invasion				0.189
Yes	14	10	4	–
No	80	42	38	–
Lymph node metastasis				0.041
Yes	22	8	14	–
No	72	44	28	–
Distant metastasis				0.313
Yes	2	1	1	–
No	92	51	41	–

### PSMA1 promotes tumor growth in vivo

To validate the influence of PSMA1 on cell growth in vivo, we subcutaneously injected PSAM1-deficient AGS cells into the nude mouse and measured tumor volume every 3 days (Fig. 7E). Tumor volumes in the PSMA1-deficient group were smaller than in the corresponding control group. Furthermore, tumor weights in the control group were heavier than that in the PSMA1-deficient group (Fig. 7F). In addition, IHC results showed the knockdown PSMA1 inhibited the expression of Ki67, YAP, and TAZ (Fig. 7G). Western blot results of tumor tissues demonstrated that knockdown PSMA1 inhibited the expression of C-Myc, PCNA, Ki67, YAP, and TAZ (Fig. 7H), which was consistent with the results in vitro. In conclusion, we demonstrated that PSMA1 promotes tumor growth on GC in vivo.

## Discussion

In this study, we verified that PSMA1 expression was upregulated in GC and associated with worse survival outcomes. Moreover, high expression was associated with high Ki67 expression, advanced TNM stage, and lymph node invasion. Further cell experiments showed the overexpression of PSMA1 significantly promoted cell proliferation, colony formation, migration, and invasion. All these results suggest that PSMA1 acts as a tumor indicator in GC and may be a valuable prognostic biomarker in GC patients. We further explore the biochemistry function for its inherent nature as a specific type of DUB, to understand the detailed mechanism between PSMA1 and malignant behaviors in GC, aiming to provide evidence for the complete PSMA1-involved regulatory network. We found that PSMA1 could stabilize TAZ through deubiquitylation, thereby upregulating the protein expression of TAZ. Such deubiquitination-mediated overexpression of TAZ could promote cell proliferation, colony formation, migration, and invasion. High expression of TAZ was an additional risk factor for PSMA1-modulated poor prognosis in GC patients. These findings demonstrate that the deubiquitinating mediator of PSMA1 promotes GC progression. Moreover, PSMA1 is a novel regulator that physically deubiquitinates and functionally stabilizes TAZ, promoting GC cell proliferation both in vitro and in vivo.

TAZ is a downstream effector of the Hippo pathway [19]. Increasing evidence has revealed that TAZ activation initiates gastric tumorigenesis in vivo and may serve as a metastatic biomarker to verify its significance in human gastric cancer [20, 21]. Both YAP and TAZ are negatively regulated by the upstream kinases LATS1/2 and MST1/2 and are subjected to ubiquitination and proteasomal degradation under physiological or pathophysiological conditions. Protein kinases are generally regarded as paradigm targets for small-molecular therapeutics, however, these kinases are difficult to be activated to show tumor-suppressive response [22]. Therefore, the external signaling nodes that converge upon and regulate TAZ can lead to better treatment options in GC patients.

As an important component of the 20S proteasome core complex, PSMA1 forms two loops and plays an integral role in the association of the 19S regulatory complex and the proteasome assembly. Some proteasomes have been found to aberrantly during carcinogenesis in various types of cancers. Studies have shown that PSMA5 is related to the pathogenic process of prostate cancer and colon cancer [23] and upregulated in lung adenocarcinoma [24]. PSMA2, PSMA3, PSMA4, PSMA6, and PSMA7 showed high expression levels, which were correlated with poor survival of breast cancer patients [25]. PSMD14 enhances hepatocellular carcinoma growth and metastasis [26]. Arlt et al. [27] demonstrated that PSMA1 mRNA levels were significantly increased in pulmonary neuroendocrine tumors relative to normal tissues. However, there are few reports on the relationship between PSMA1 and GC.

As TAZ is a crucial factor that initiates gastric carcinogenesis, it has been demonstrated that the expression of TAZ is post-translationally regulated through the ubiquitin-proteasome pathway. Although some DUB of TAZ, such as USP1, USP 10, and OTUB2, had been demonstrated to regulate its ubiquitination and stabilization [17, 18, 28]. Commonly, multiple deubiquitinating enzymes targeted the same substrate to regulate its stability under different circumstances. Such as, the snail is deubiquitinated and stabilized by PSMD14, DUB3, and USP26 [29–31]. Ubiquitination of P53 is downregulated by deubiquitinating enzymes USP10 and TRIM31 [32–34]. Therefore, we hypothesize that TAZ stability and ubiquitination are also regulated by multiple deubiquitinating enzymes. To our knowledge, this is the first study to detect the expression and function of PSMA1 in GC. In this study, we found that PSMA1 is a specific deubiquitinase of TAZ which could significantly increase TAZ stability. The major deliverables for protein ubiquitination modification are the linkage of multiple ubiquitin chains with the protein substrate. The K48-linked Ub chains generally serve as an inducer of proteasome-mediated proteolysis [35], while the main function of K63-linked Ub chains is nondegradative and, for example, activates protein kinase cascades [36]. K11-linked Ub chains constitute an alternative degradation signal used during cell-cycle progression [37]. The other types (K6, K27, K29, and K33) are elusive [38]. In the present study, our experimental data revealed that PSMA1 could regulate the stability of TAZ by relieving their Lys 27-linked and Lys 48-linked ubiquitination. Together, these findings help to fully elicit the relationship between the molecular conformation and function polymorphism of PSMA1. Moreover, it advances the completion of the PSMA1-mediated mechanism network, specifically in terms of deubiquitination modification. Here, our findings showed that knockdown PSMA1 downregulation proliferation markers PCNA and C-Myc, and reversed results were also observed after overexpression of PSMA1. Such events can be reversed by overexpression of TAZ, suggesting that the regulation of the proliferation process by PSMA1 is dependent on TAZ. In addition, we found that TAZ was highly expressed in GC tissues compared with adjacent normal tissues. Subsequent survival analysis suggested that high expression of TAZ predicted poor survival in GC patients. These findings indicate a potential relationship between TAZ expression and an aggressive phenotype in GC, consistent with previous results [20, 22, 39]. We found that deubiquitination modification was an effective regulator for maintaining the protein levels of TAZ, thereby establishing their foundation to drive GC cell proliferation.

## Conclusion

**Fig. 8:**
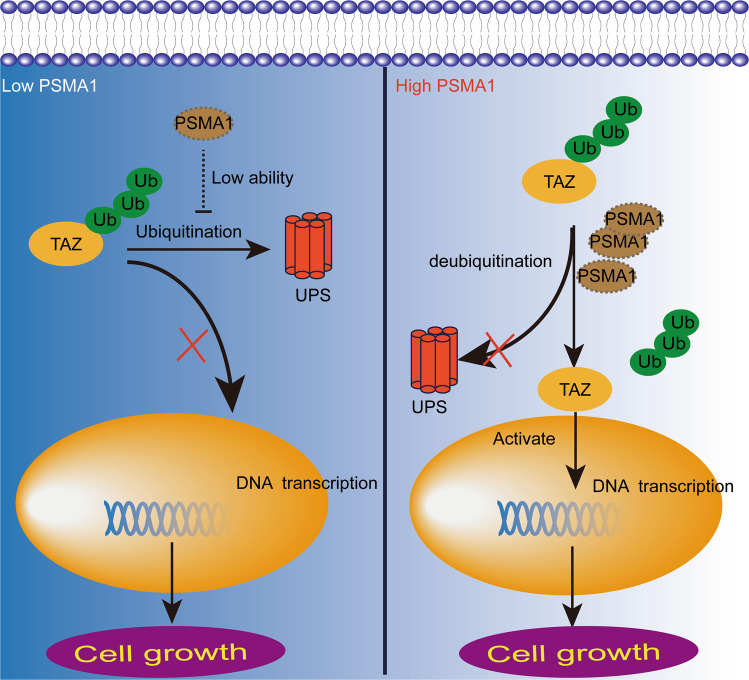
Scheme for the regulatory mechanism of PSMA1 on TAZ.

In summary, the upregulated PSMA1 stabilized TAZ protein to promote GC proliferation (Fig. 8). PSMA1 was highly expressed in GC and correlated with poor survival prognosis. Upregulation of PSMA1 facilitated proliferation, colony formation, migration, and invasion. Further studies showed that PSMA1 directly interacts with TAZ and then inhibited the K27- and K48-linked ubiquitination of TAZ to stabilize and activate TAZ. Upregulate of TAZ reversed the inhibitory effects of PSMA1 on the growth of GC cells in vitro and in vivo. PSMA1 is associated with the TAZ protein and inhibits its polyubiquitination and proteasome-dependent degradation in GC cells. This founding indicated that PSMA1 may serve as a novel prognostic predictor and therapeutic target for GC.

## Materials and methods

### Cell culture, antibodies, and reagents

GC cells (AGS, HGC-27, BGC-823, MKN-45, MKN-74, and SGC-7901) and human gastric mucosal epithelial cells (GES-1) were purchased from the Beijing Institute of Cancer Research (Beijing, China). HEK-293T cells were purchased from Procell Biotech (Wuhan, China). GC cells and HEK-293T cells were maintained in Dulbecco’s Modified Eagle Medium (DMEM) (Gibco Company, USA) containing 10% fetal bovine serum (FBS) 100 unit/mL penicillin and 100 g/mL streptomycin in a humidified atmosphere at 37 °C and 5% CO2. The GES-1 cell was cultured in RPMI-1640 medium (Gibco Company, USA) mixed with 10% FBS. In this experiment, antibodies used as follows, PSMA1(ab109530, Abcam), PSMA1(sc-166073, Santa Cruz), YAP (#4912, CST), YAP (13584-1-AP, Proteintech), TAZ (#83669, CST), TAZ (23306-1-AP, Proteintech), Ki67 (ab1667, Abcam), ACTIN (20536-1-AP, Proteintech), FLAG (20543-1-AP, Proteintech), MYC (#2276, CST), HA (51064-2-AP, Proteintech), PCNA (10205-2-AP, Proteintech), and C-Myc (10828-1-AP, Proteintech).

### Patient samples, tissue microarray, and immunohistochemistry

One hundred and two samples including SG (*n* = 24), IM (*n* = 24), DYS (*n* = 21), GC (*n* = 33) were acquired from the pathology department of the first affiliated hospital of Nanchang University. GC sample and corresponding normal gastric tissues were used to construct a TMA (Outdo Biochip, Shanghai, China). IHC staining assay was used to detect PSMA1 and TAZ expression. The IHC score was independently by two pathologists for both staining intensity and the percentage of positively stained cells. The scoring of tissue slides was calculated based on intensity and the proportion of positive cells. The intensity of the cancer cell staining was scored as 0 for negative staining, 1 for weak staining (light yellow), 2 for moderate staining (yellow), and 3 for intense staining (yellowish brown); the proportion of positive cells was scored as 0 (1–5%), 1 (6–25%), 2 (26–50%), 3(51–75%), and 4 (76–100%). The results were assessed by two pathologists blindly and divided into three groups: high expression (7–9), moderate expression (4–7), and low expression (0–3). This study was approved by the institutional human ethics committee of the First affiliated hospital of Nanchang University.

### Plasmid construction, small interfering RNA, and Lentivirus transfection

The empty vector, Flag-PSMA1, PSMA1 domain deletion mutant plasmids, TAZ domain deletion mutant plasmids, K214R mutant for TAZ and Ub-K6, Ub-K11, Ub-K27, Ub-K29, Ub-K33, Ub-K48, Ub-K63 encoding Flag, Myc or HA tags were purchased from Hanbio Tech (Shanghai, China). Wild-type TAZ plasmids were purchased from Genechem Technology (Shanghai, China). For RNA interference studies, GC cells were transfected with predesigned small interfering RNAs (siRNAs) at a final concentration of 25 nM. The siRNAs of PSMA1 sequences were as follows: siRNA1, sense (5′-3′) CCAUGUUGACAACCAUAUUTT, antisense (5′-3′) AAUAUGGUUGUCAACAUGGTT; siRNA2, sense (5′-3′) GCUGAUGCUAGACUGUUAUTT, antisense (5′-3′) AUAACAGUCUAGCAUCAGCTT; and siRNA3, sense (5′-3′) GCCUGUGUCUCGUCUUGUATT, antisense (5′-3′) UACAAGACGAGACACAGGCTT. Lentivirus of shPSMA1 was obtained from Wuhan favor biotechnology service, and the shRNA sequences were GCCUGUGUCUCGUCUUGUATT. The cells were transfected with plasmid or siRNA duplexes using Lipofectamine 2000 (Invitrogen, Carlsbad, CA, USA) according to the manufacturer’s instructions. The effect of target protein expression was evaluated by western blotting 48 h post-transfection.

### Real-time qRT-PCR

Total RNA from tissues was lysed with TRIZOL reagent (TransGen Biotech, Beijing) and extracted with chloroform and isopropyl alcohol. Then, the RNA concentration was measured with a NanoDrop 2000 spectrophotometer (Thermo Scientific, Wilmington, DE, USA). cDNA synthesis was synthesized of reagents purchased from TransGen Biotech following the manufacturer’s protocol. The real-time PCR was performed by using the reagents (Yeasen Biotechnology, Shanghai, China). The forward and reverse PCR primers for PSMA1 were 5′-AACAAGGTTCAGCCACAGTTG-3′ and 5′-ACACAGGCAGTGGTCTATCG-3′, and for TAZ were 5′-GTCCTACGACGTGACCGAC-3′ and 5′-CACGAGATTTGGCTGGGATAC-3′, for ACTIN were 5′-GCGTGACATTAAGGAGAAGC-3′, and 5′- CCACGTCACTTCATGATGG-3′.

### Co-immunoprecipitation and western blot analysis

Co-IP assay was conducted to the interaction between PSMA1 and TAZ. AGS and BGC-823 cells were seeded in 6-wells plates and then scraped with RIPA lysis buffer. To precipitate the target proteins, 500 μl of post-centrifuged lysates was incubated with 1 μl primary antibody followed by incubation with 50 μl protein A/G agarose (Santa Cruz Biotechnology, Texas, USA) and 500 μl of phosphate-buffered saline (PBS) was mixed and incubated together for 2 h. Next, the cellular proteins were incubated with corresponding antibodies overnight at 4 °C. The next day, the cells were centrifuged at 7500 rpm at 4 °C and washed twice with PBS containing protein inhibitors. The immunoprecipitated proteins were eluted with SDS-PAGE loading buffer by boiling for 10 min. Western blotting was performed according to related protocol. The proteins in the lysate were separated by SDS-PAGE and then transferred to nitrocellulose membranes (Millipore). The membranes were blocked with 5% BSA (Solarbio Life Science, Beijing, China) and incubated with corresponding antibodies at 4 °C overnight. The next day incubated with horseradish peroxidase-conjugated secondary antibodies (TransGen Biotech, Beijing) for 1 h at room temperature.

### Mass spectrometry

AGS cells transfected with siPSMA1 and siCtrl were collected and transferred for further analysis. After 48 h, all protein was extracted in radioimmunoprecipitation assay (RIPA) buffer (Solarbio Life Science, Beijing, China) mixed with protease inhibitor on ice-cold plates. Then, the products were performed liquid chromatography-tandem mass spectrometry (LC–MS/MS) (24600, Thermo) analysis. TMT was performed and analyzed by Luming Biotechnology (Shanghai, China). A fold change (FC) > 2 or < 0.5 with a P < 0.05 was considered as significant for differential expression.

### Cell proliferation, colony formation, and transwell assay

CCK-8 assay was used to detect the cell proliferation, the cells (2000) were seeded into 96-wells plates. The next day, the cells were transfected with PSMA1 siRNA or PSMA1 overexpression plasmid. Then, cell viability was detected by a CCK-8 (Solarbio Life Science, Beijing, China) assay at 450 nm. The colony formation assay was performed to detect the cell proliferation, and the cells (800) were plated in 6-wells in each group and cultured. After 2 weeks, cell colonies in the plates were fixed with 4% paraformaldehyde and stained with 1% crystal violet. The numbers of cell colonies were counted using Image J. The transwell assay was used to detect the migration and invasion abilities of gastric cancer cells and evaluated by coating with or without Matrigel Transwell (Corning, USA). 1.5 × 10^4^ AGS cells and 3 × 10^4^ BGC-823 cells in 200 μl serum-free medium were seed into the upper chamber with or without pre-coated Matrigel, and containing 20% FBS medium was moved into the lower chamber. After 48 h, the migratory or invasive cells were fixed with 4% paraformaldehyde for 20 min and stained with 1% crystal violet for 30 min. The number of invading cells was counted in ten random visual fields per chamber under a microscope (Olympus, Tokyo, Japan).

### Immunofluorescence staining and confocal laser scanning microscopy

GC cells were incubated in chamber slides for 24 h, fixed with 4% paraformaldehyde for 20 min, and permeabilized with 0.1% Triton X-100 and 0.1 g BSA for 1 h. Next, cells were seeded with anti-PSMA1 (sc-166073, Santa Cruz) and anti-TAZ (23306-1-AP, Proteintech) overnight at 4 °C. Finally, the cells were incubated with IgG fluorescent secondary antibody (488, ab150113, 594, ab 150077, Abcam), and nuclei were stained with DAPI. Immunofluorescence intensity was examined via confocal fluorescence microscopy at wavelengths of 488 and 594 nm.

### Protein half-life assay

GC cells were transfected with PSMA1 siRNA and treated with 20 μM CHX for the times indicated in the figure legends. Western blotting was used to determine the protein level of TAZ at the indicated times.

### Deubiquitination assay

HEK-293T cells were transfected with HA-Ub and Myc-TAZ with or without Flag-PSMA1 for 48 h. After the cells were treated with 20 μM of the proteasome inhibitor MG132 for 6 h, cell lysates were prepared with cell lysis buffer and immunoprecipitated with the indicated antibodies (anti-Myc) on protein A/G beads (Santa Cruz Biotechnology, Texas, USA) overnight. The beads were then washed and boiled in SDS loading buffer. Immunoprecipitated protein complexes were assessed by western blotting with anti-HA.

### Animal study

Six-week-old BALB/c nude female mice were purchased from Vital River (Beijing, China). Twenty female thymus-null BALB/c nude mice were divided into two groups. AGS cells (4 × 10^6^ cells) were transfected with PSMA1 shRNA or negative control and injected intravenously via the subcutaneous into the respective groups. Approximately seven days later, tumor size was measured using a vernier caliper. Tumor volumes were calculated using the formula *V* = ½ (length × width^2^). These mice were sacrificed one month after injection, half of the tumor tissue was used to extract protein, and the residue was fixed in 10% neutral formalin, embedded in paraffin, and cut into 3-μm thick for further IHC staining. All animal experiments were approved by the Animal Care and Use Committee of Nanchang University.

## Supplementary information

Supplementary Figure legends

Original Data File

Supplementary Figure 1

Supplementary Figure 2

Supplementary Figure 3

Supplementary Figure 4

## Data Availability

The data supporting the conclusions of this article are provided in this article and the additional files. In addition, all data from this study can be obtained from the corresponding author upon reasonable request.
