# Supplementary material for: PSMA1 mediates tumor progression and poor prognosis of gastric carcinoma by deubiquitinating and stabilizing TAZ
Source: Cell Death Dis. 2022 Nov 23;13(11):989. doi: 10.1038/s41419-022-05417-0 (PMC9691733; doi:10.1038/s41419-022-05417-0)
Supplement: Supplementary file 1 — Supplementary Figure legends [file 41419_2022_5417_MOESM1_ESM.docx]

**Supplementary Figure 1. PSMA1 regulation imposed no effect on TAZ mRNA. A, B** PSMA1 imposed little effect on the mRNA levels of TAZ.

**Supplementary Figure 2. K214 ubiquitination of TAZ identified by mass spectrometry. A** Mass spectrometry of TAZ protein.

**Supplementary Figure 3. The function of TAZ-K214R. A** The representative images of colony formation and its statistical results of AGS cells in the indicated group. **B** CCK-8 assay was used to detect the proliferation of AGS cells in the indicated group. **C** Transwell assay results for testing the migration and invasion ability of AGS cells in the indicated group, and their statistical results. Scale bar, 100 μm.

**Supplementary Figure 4. Representative images of IHC staining of TAZ in normal and gastric cancer tissues. A** IHC staining for TAZ in gastric cancer tissue and normal tissue. **B** IHC score analyzed the expression levels of TAZ in GC tissues compared with the corresponding adjacent normal tissues.
