# Supplementary material for: PSMA1 mediates tumor progression and poor prognosis of gastric carcinoma by deubiquitinating and stabilizing TAZ
Source: Cell Death Dis. 2022 Nov 23;13(11):989. doi: 10.1038/s41419-022-05417-0 (PMC9691733; doi:10.1038/s41419-022-05417-0)

Figure 1

1B β-actin

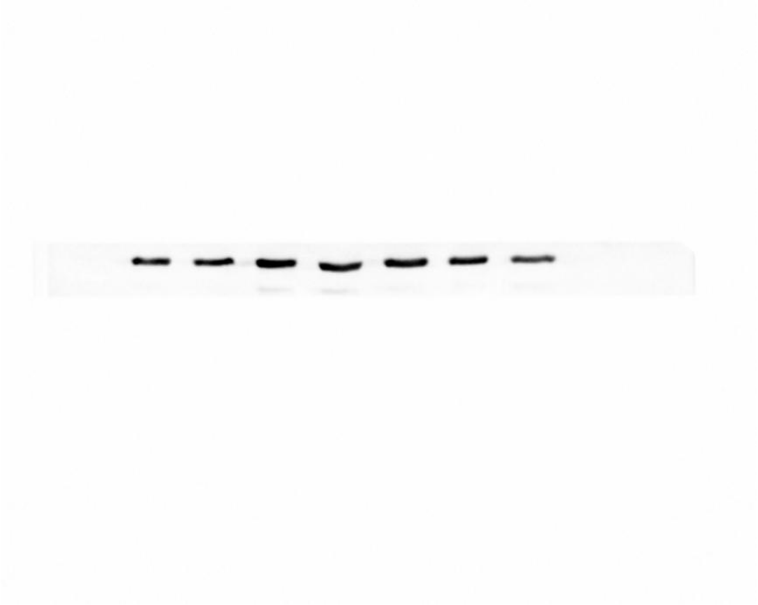

1B PSMA1

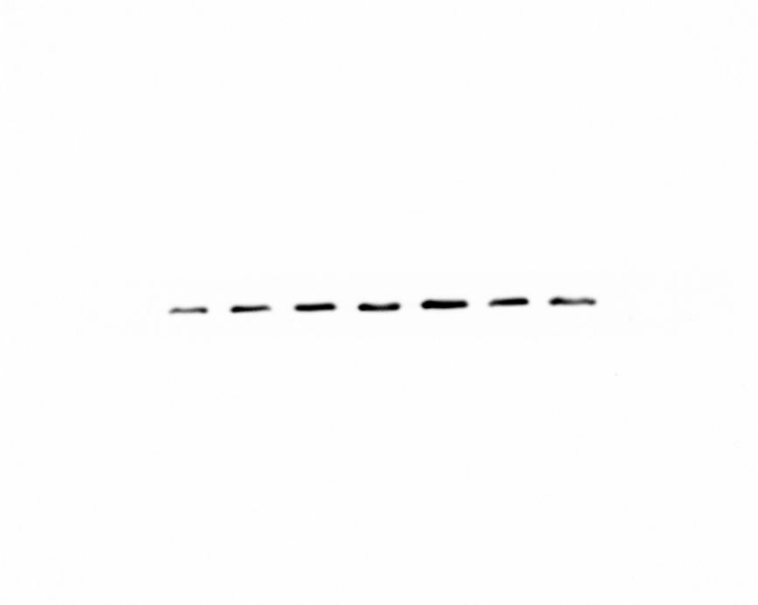

Figure 2

2E AGS, BGC823 β-actin

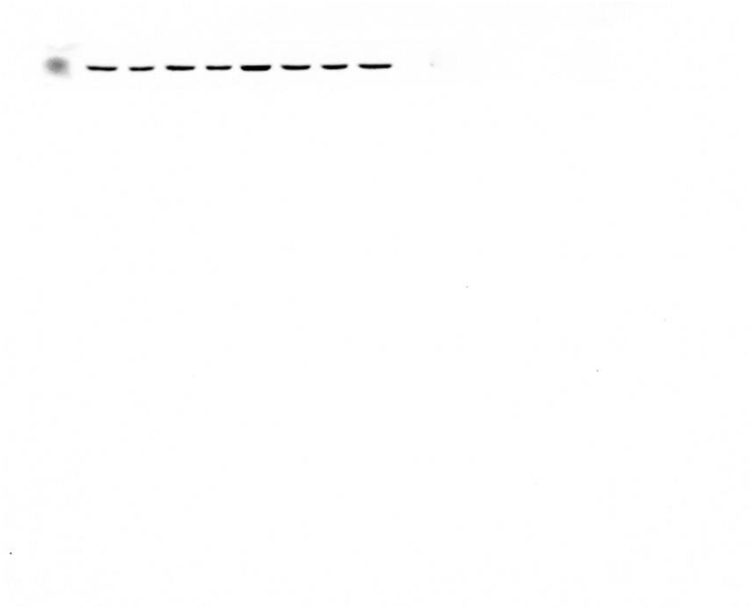

2F AGS, BGC823 β-actin

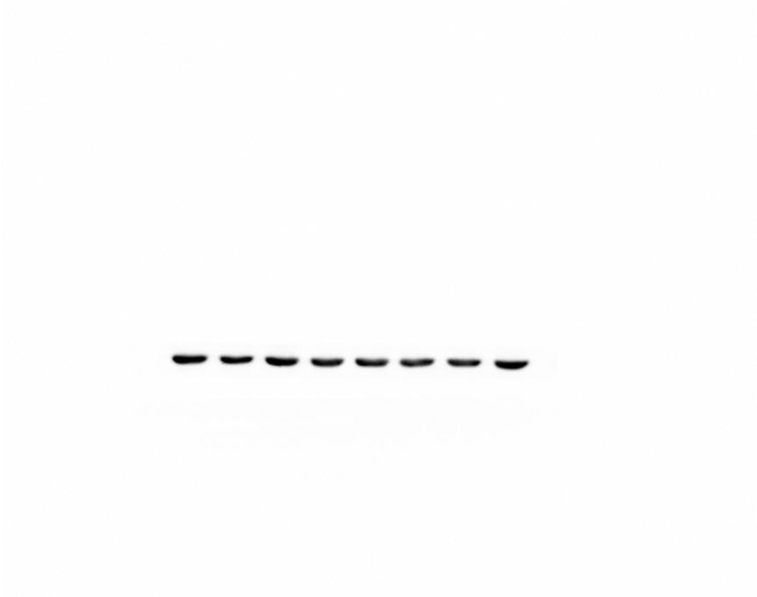

2E AGS, BGC823 β-actin

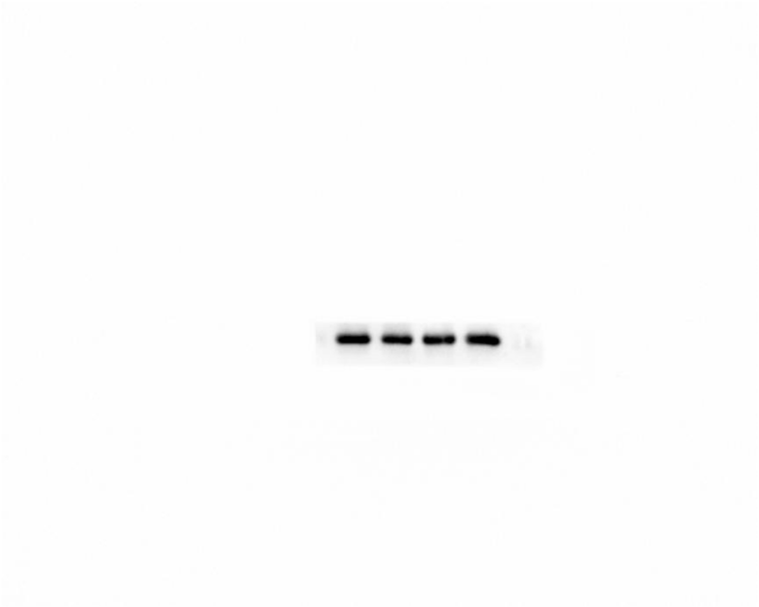

2F AGS, BGC823 β-actin

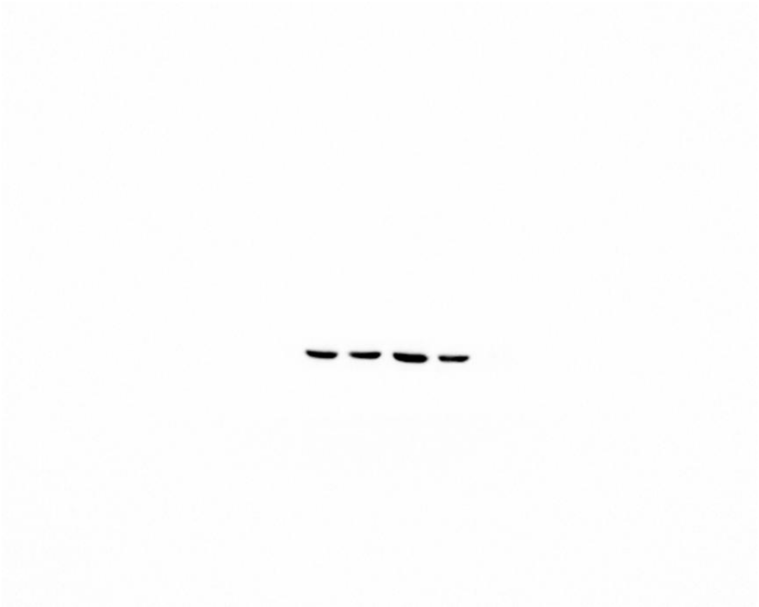

2E AGS, BGC823 PSMA1

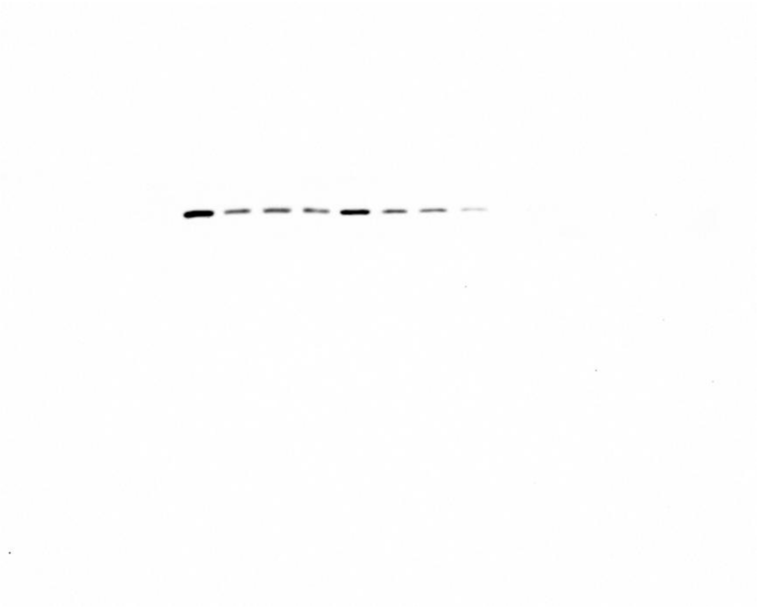

2E AGS, BGC823 TAZ

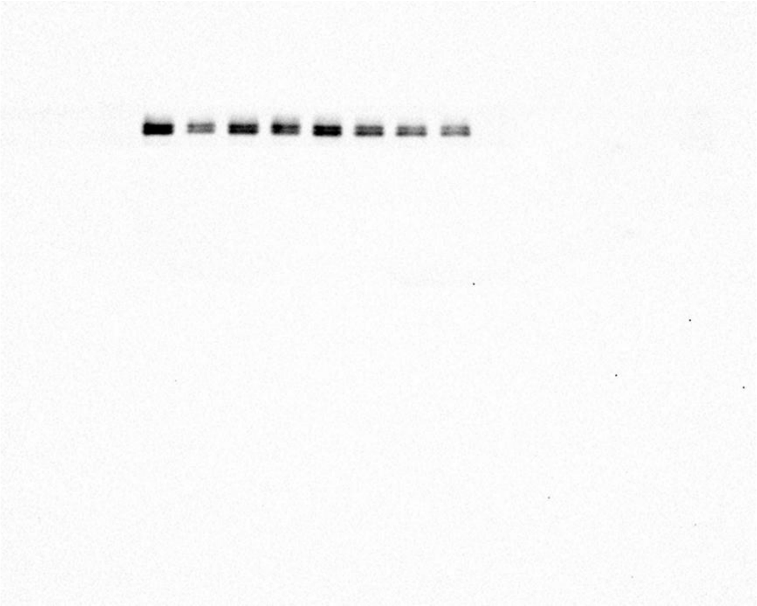

2F AGS, BGC823 PCNA

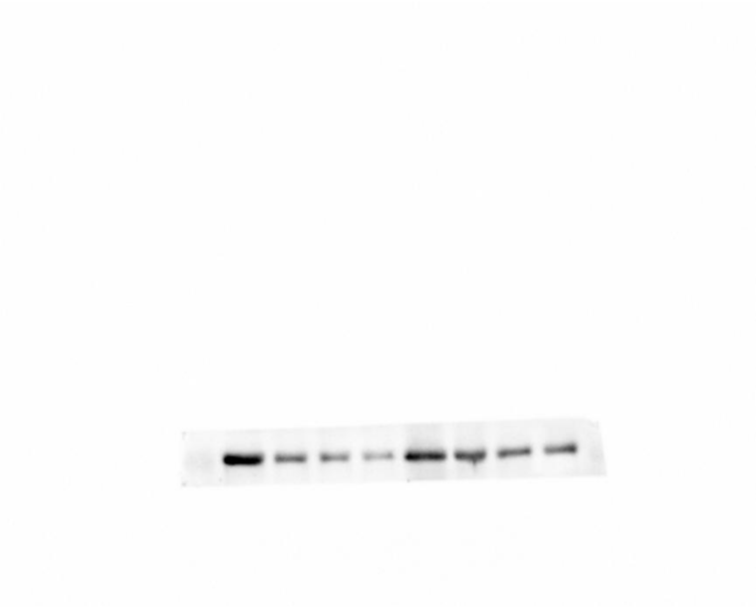

2E AGS, BGC823 YAP

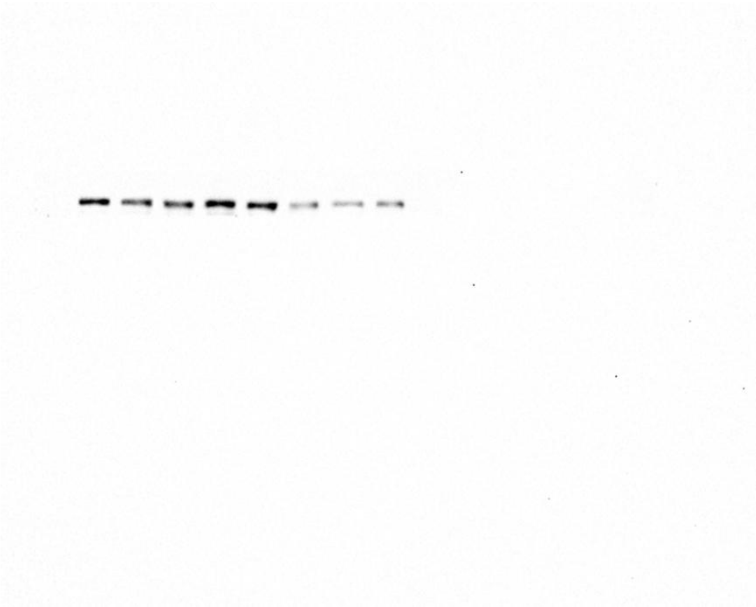

2F AGS, BGC823 C-Myc

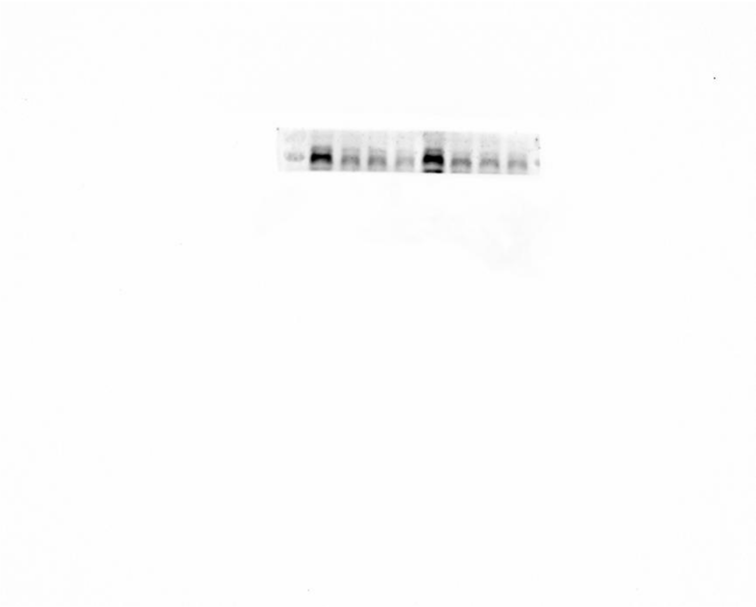

2F AGS, BGC823 PSMA1

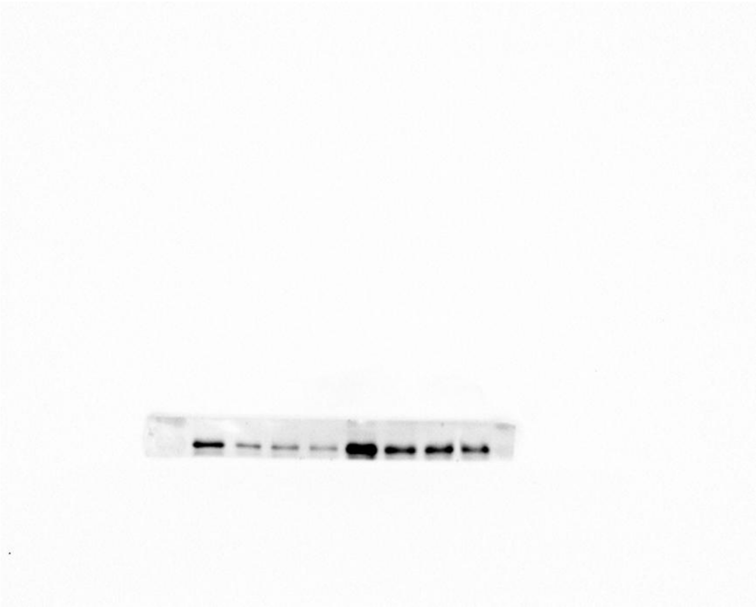

2E AGS, BGC823 TAZ

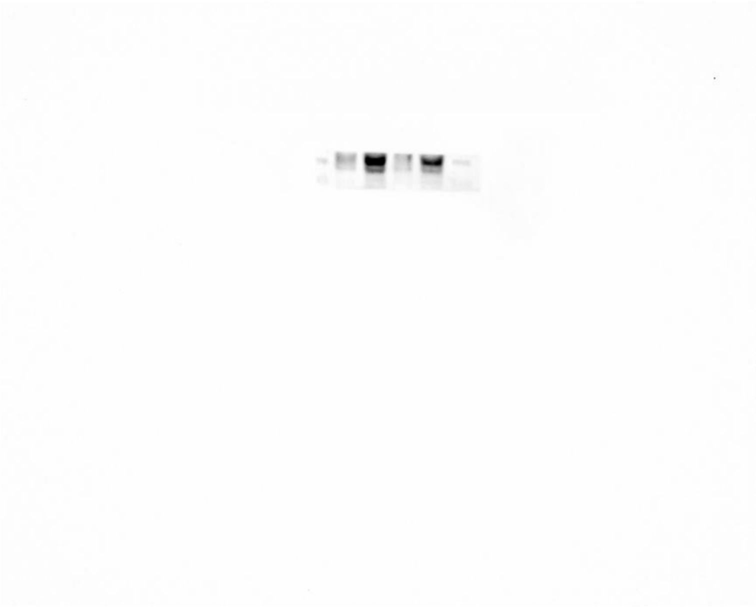

2E AGS, BGC823 YAP

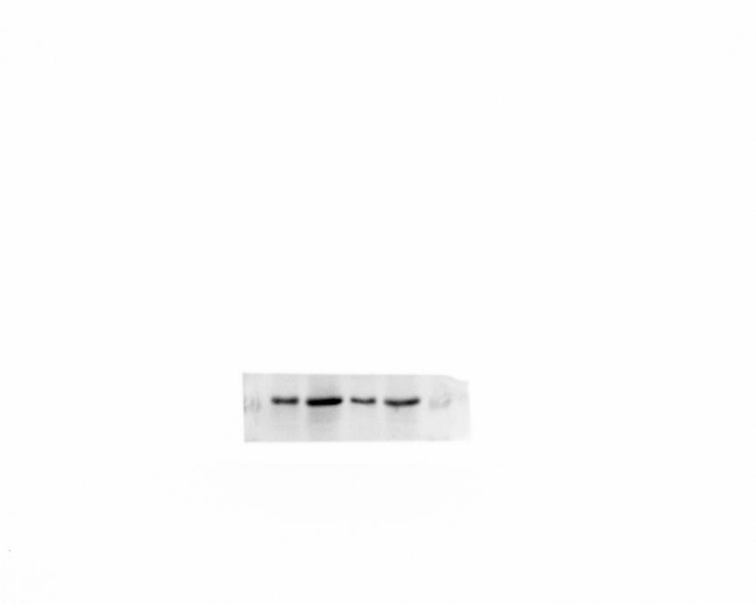

2E AGS, BGC823 PSMA1

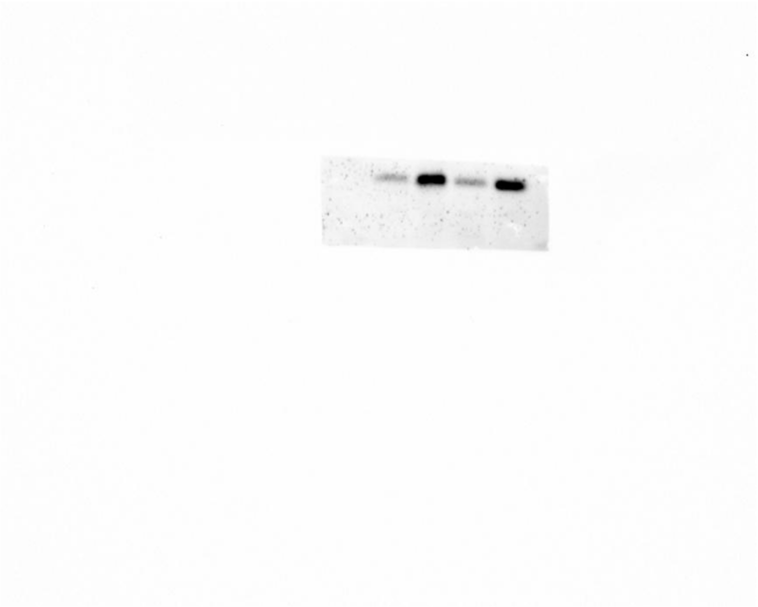

2F AGS, BGC823 PSMA1

2F AGS, BGC823 C-Myc

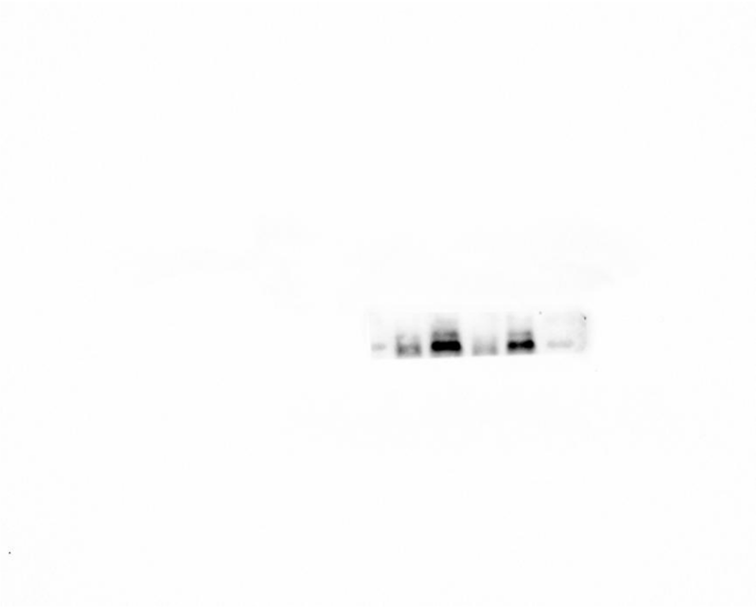

2F AGS, BGC823 PCNA

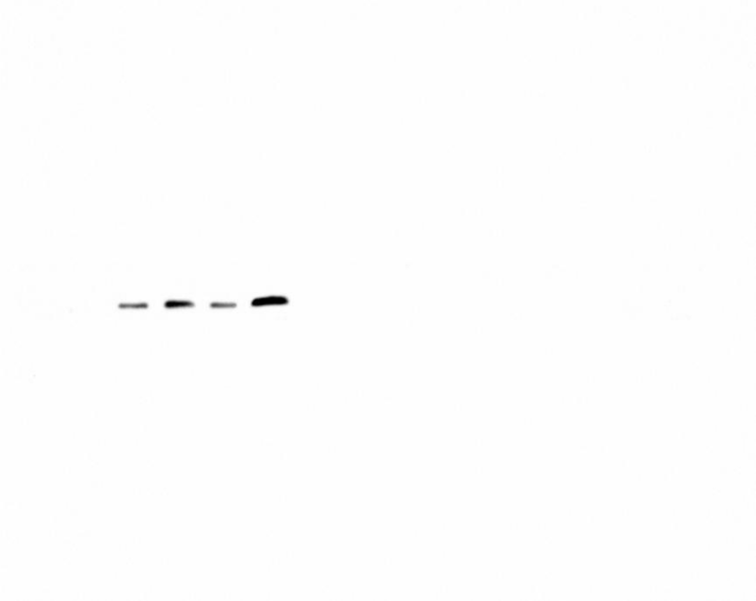

Figure 3

3A AGS PSMA1

3A AGS β-actin

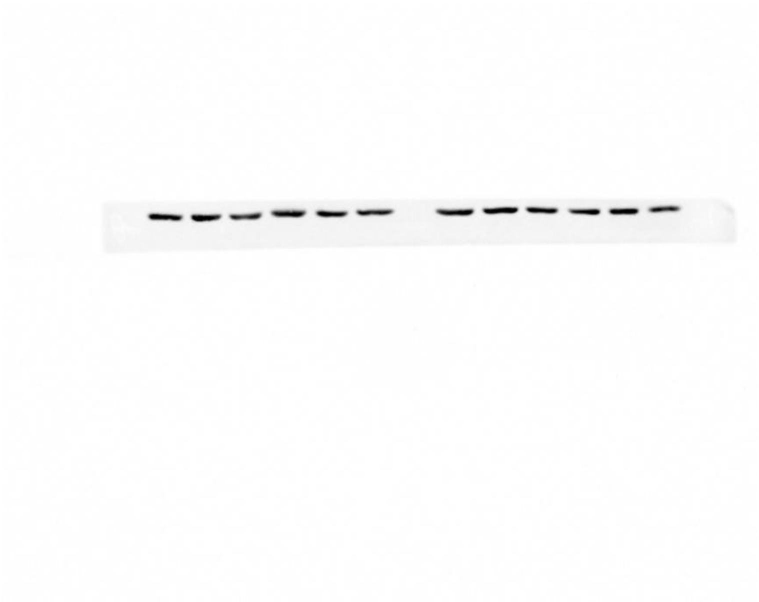

3A AGS TAZ

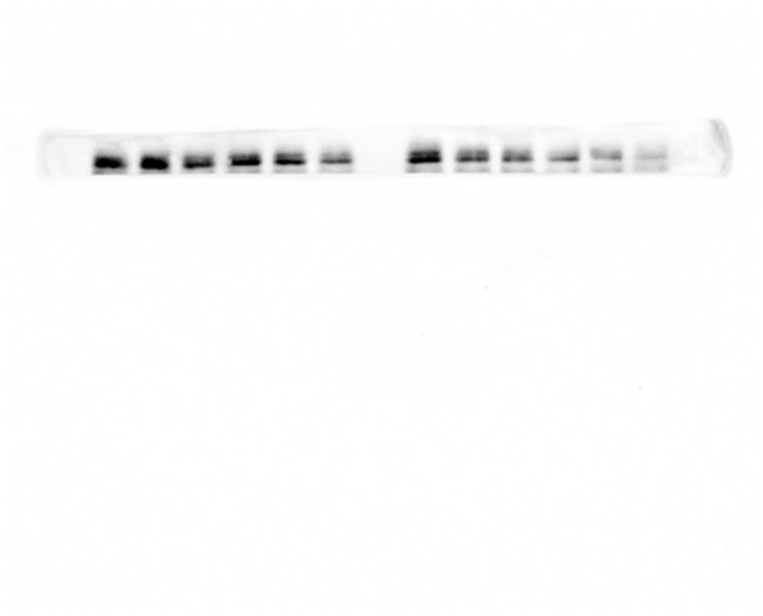

3A BGC-823 β-actin

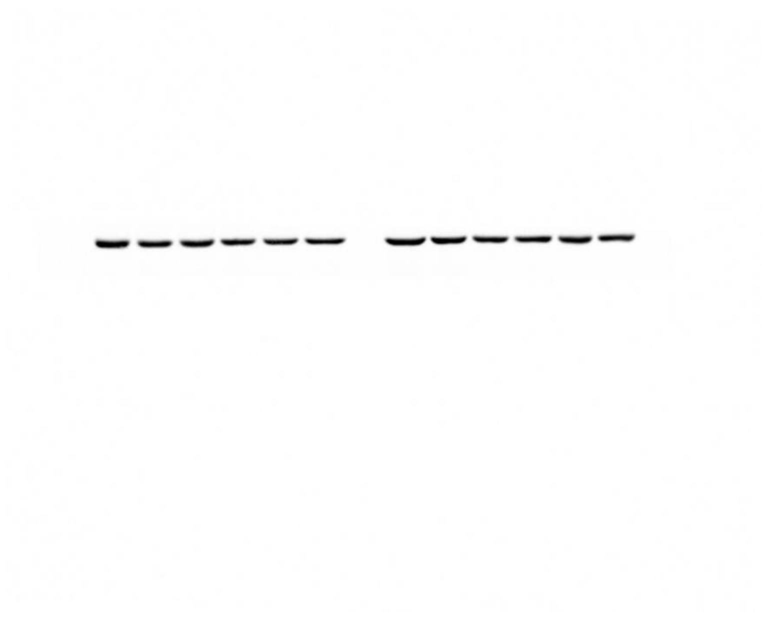

3A BGC-823 PSMA1

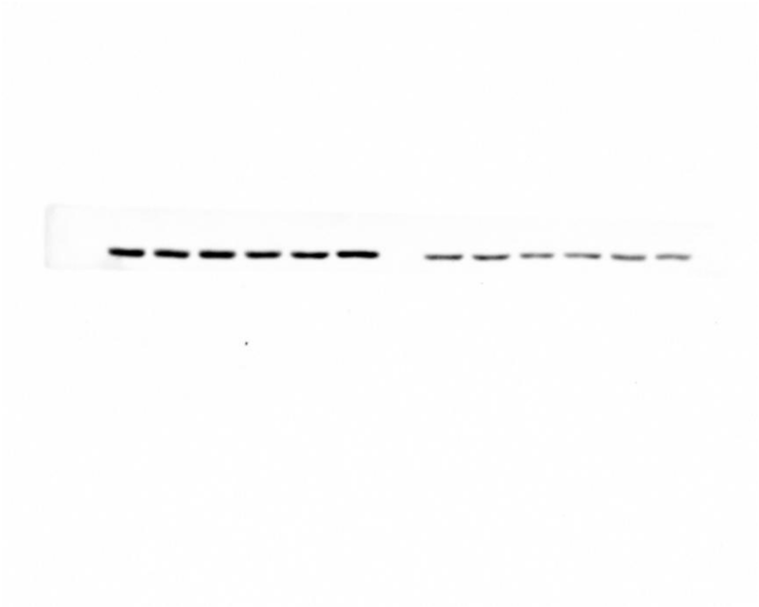

3A BGC-823 TAZ

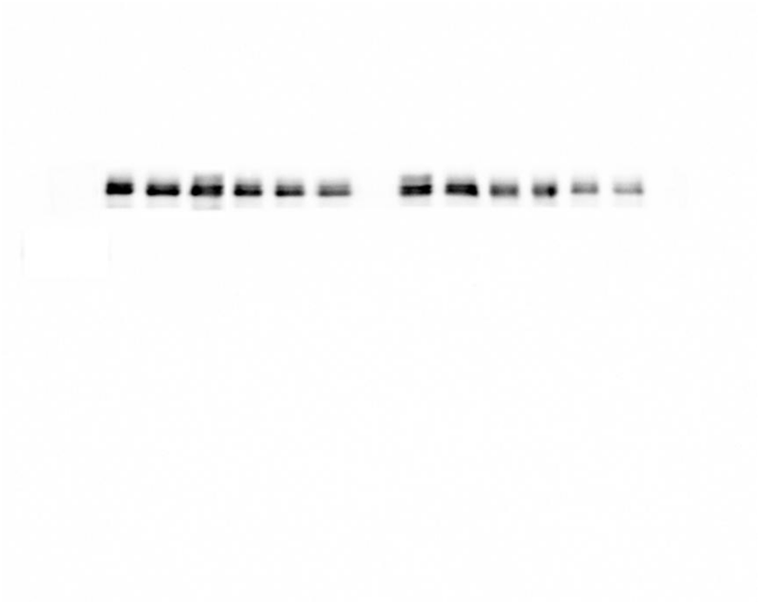

3B AGS BGC-823 β-actin

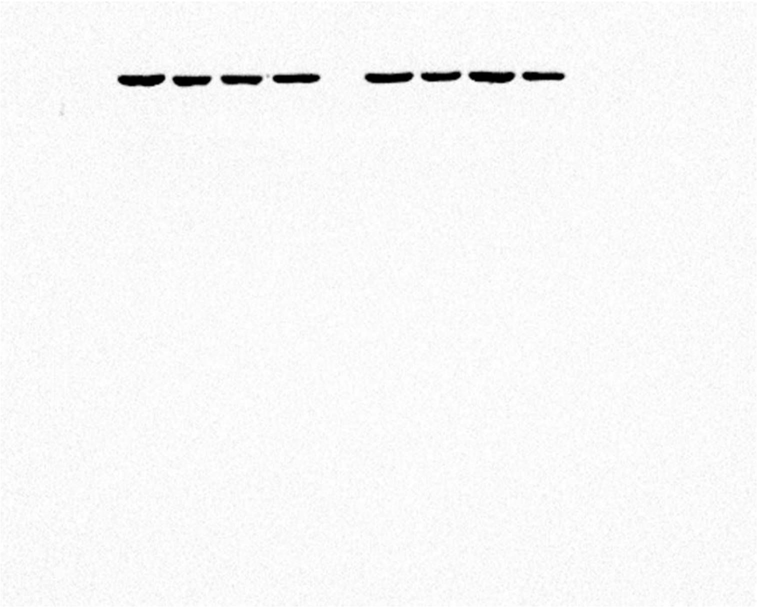

3B AGS BGC-823 PSMA1

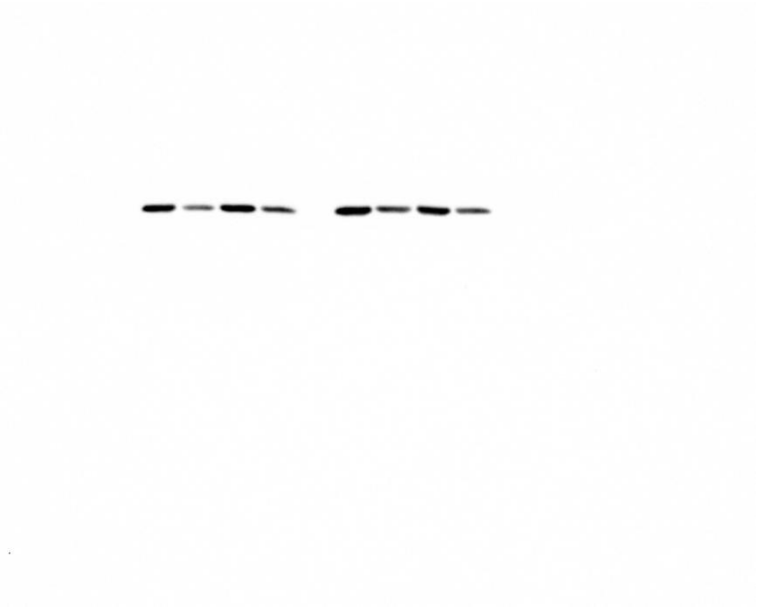

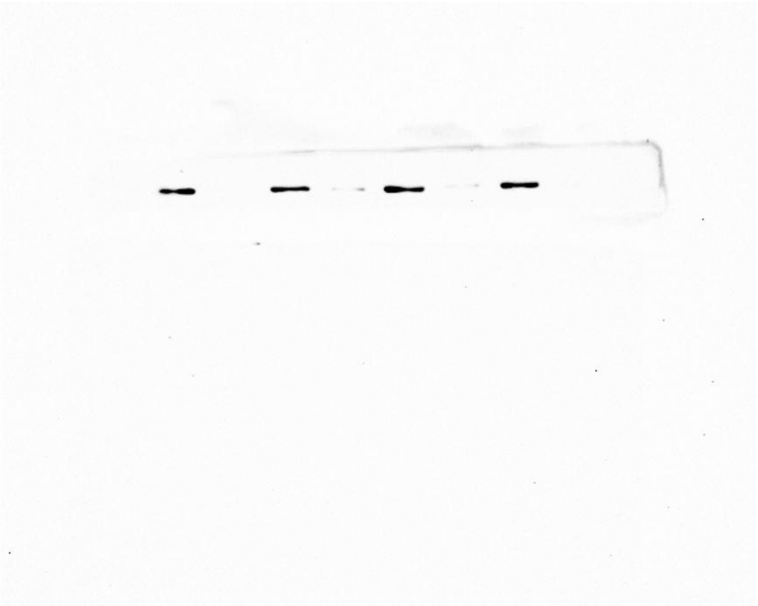

3B BGC-823 TAZ

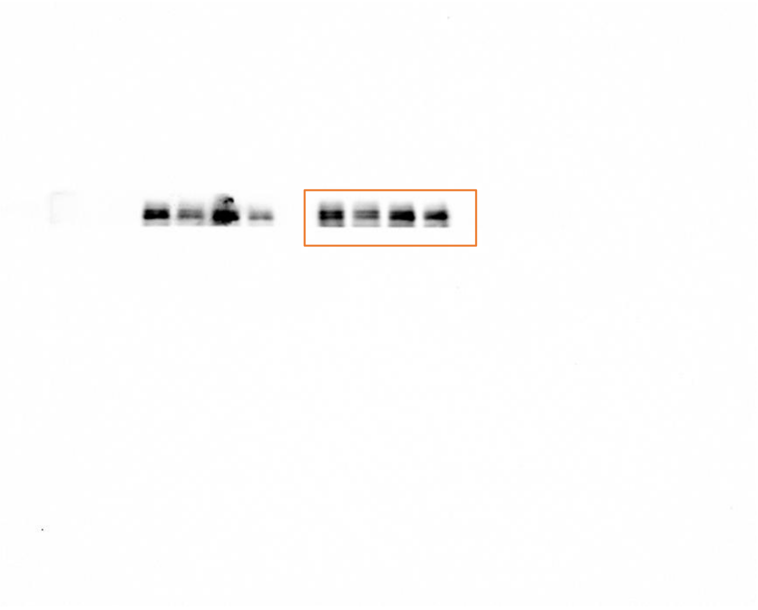
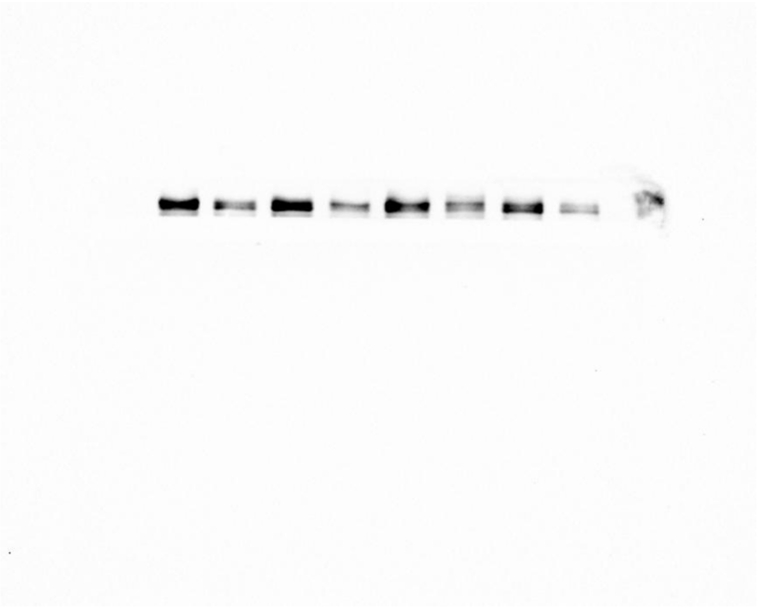

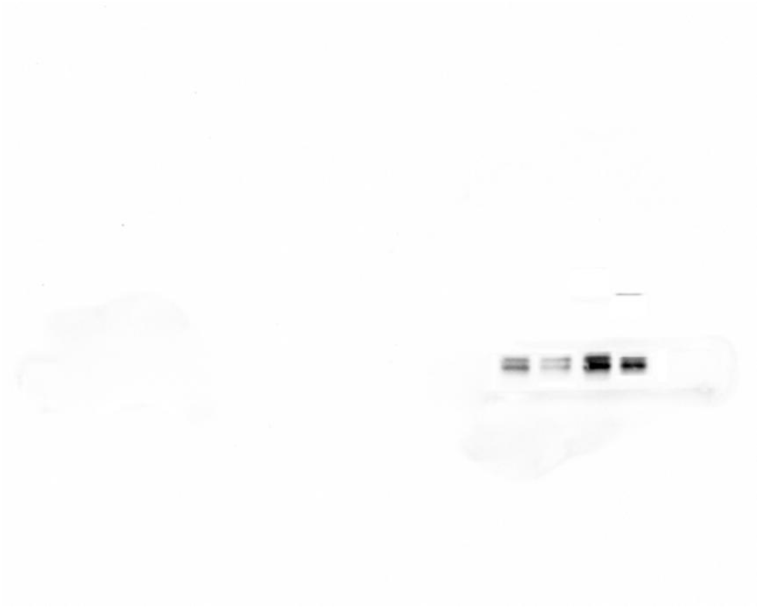

3C BGC-823 β-actin

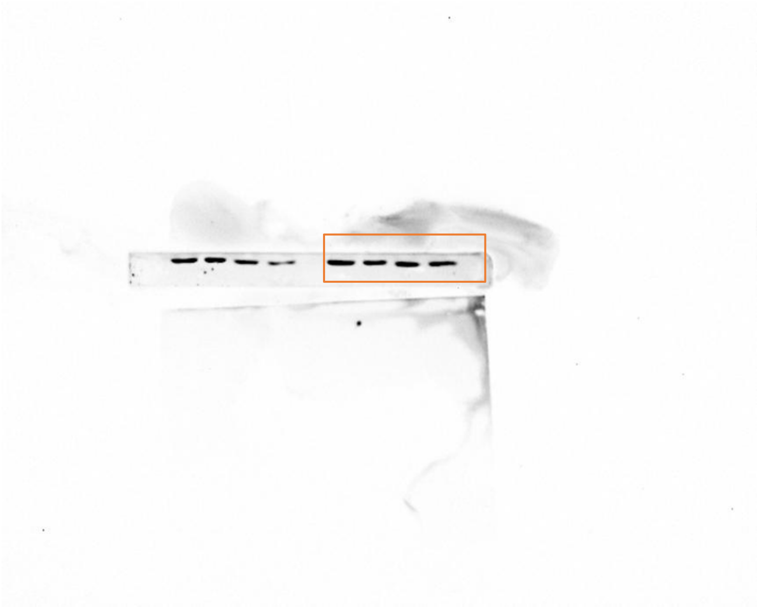

3C AGS β-actin

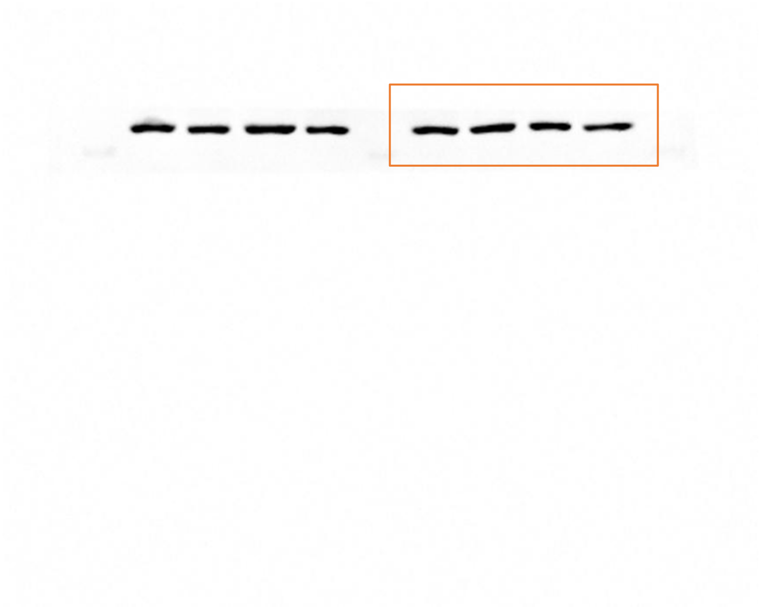

3C BGC-823 TAZ

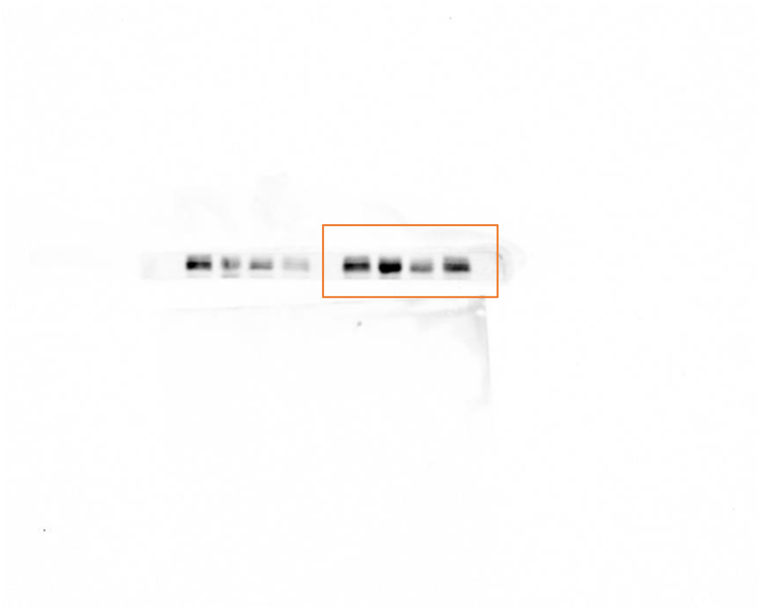

3C AGS TAZ

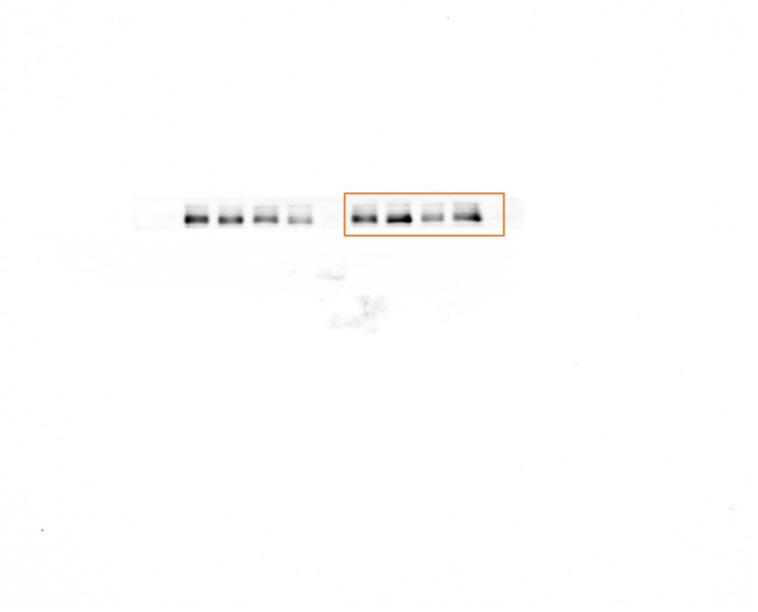

3D HA

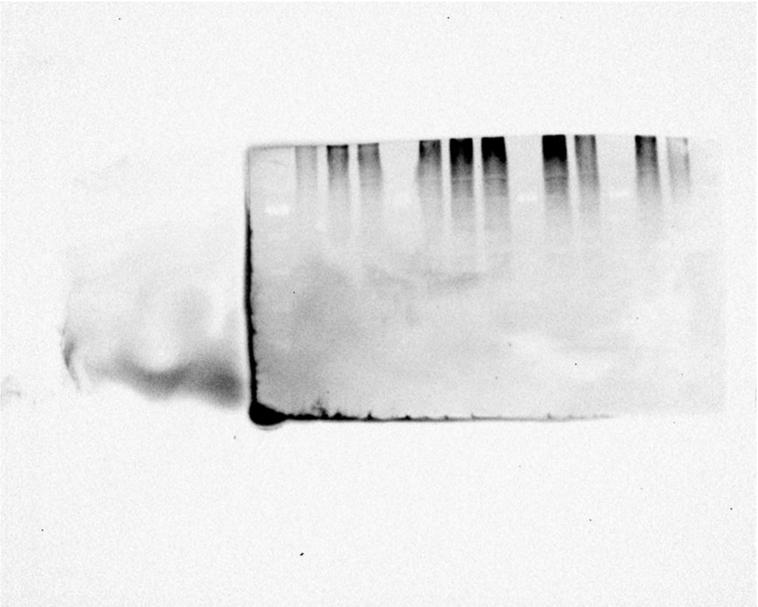

3D IP Myc

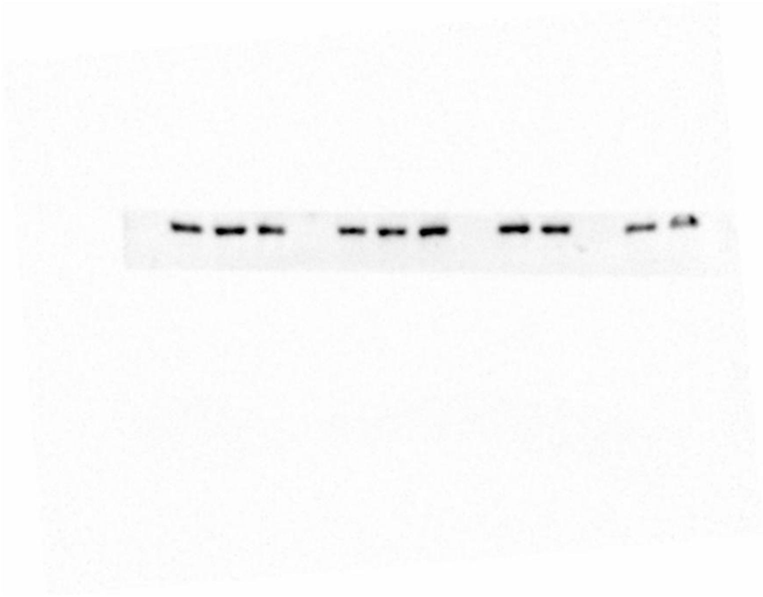

3D IB Myc

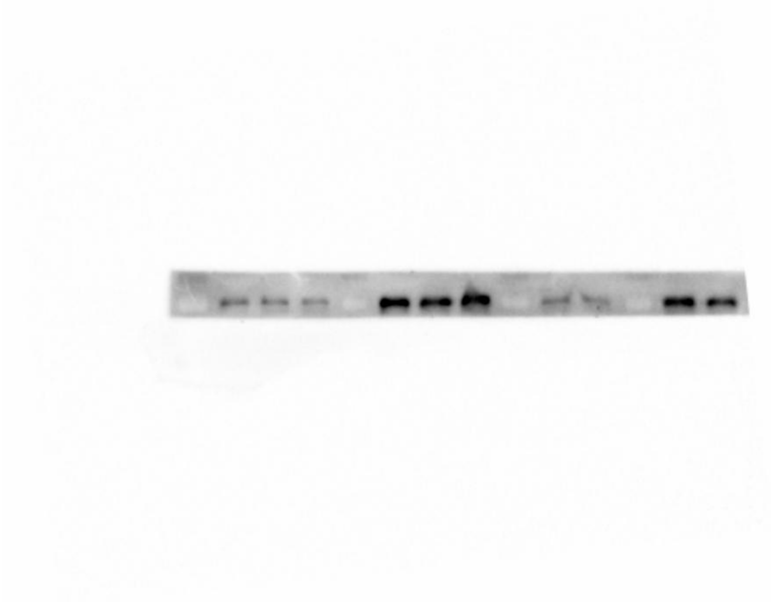

3D β-actin

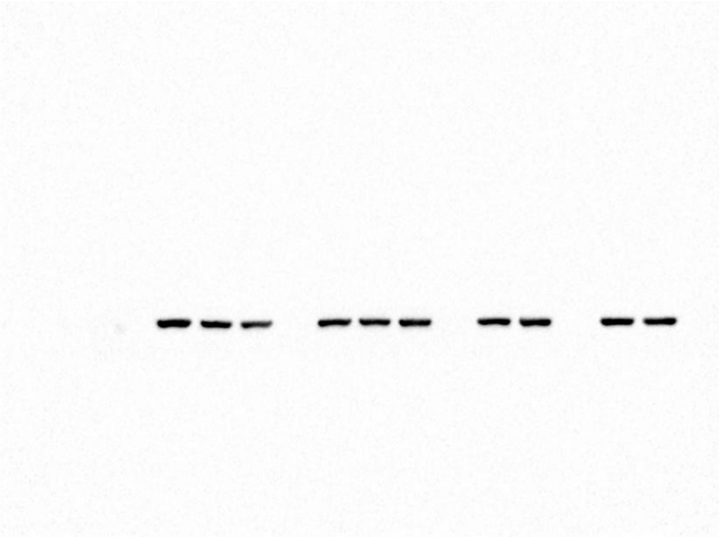

3D PSMA1

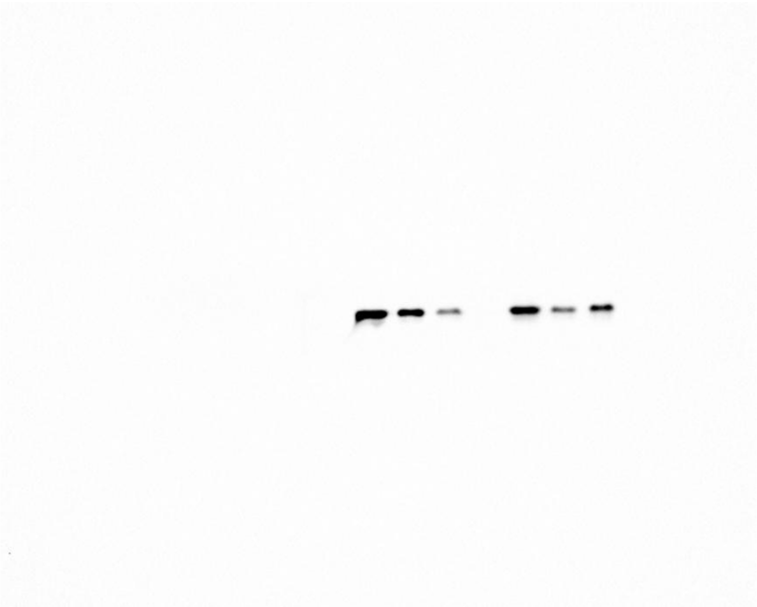

3D Flag

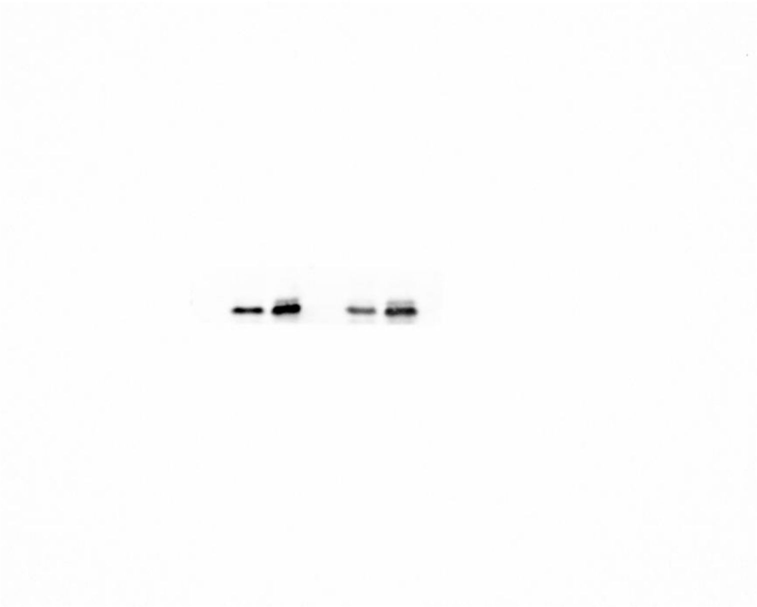

Figure 5

5A PSMA1

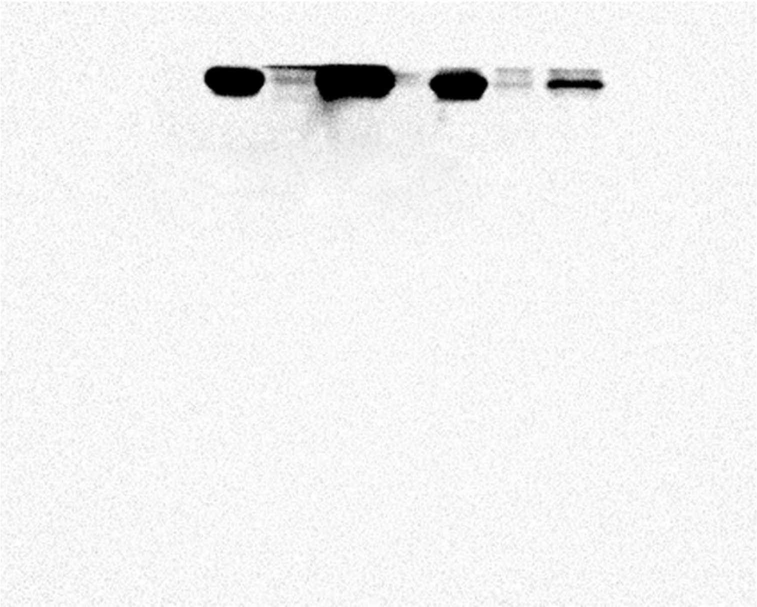

5A TAZ

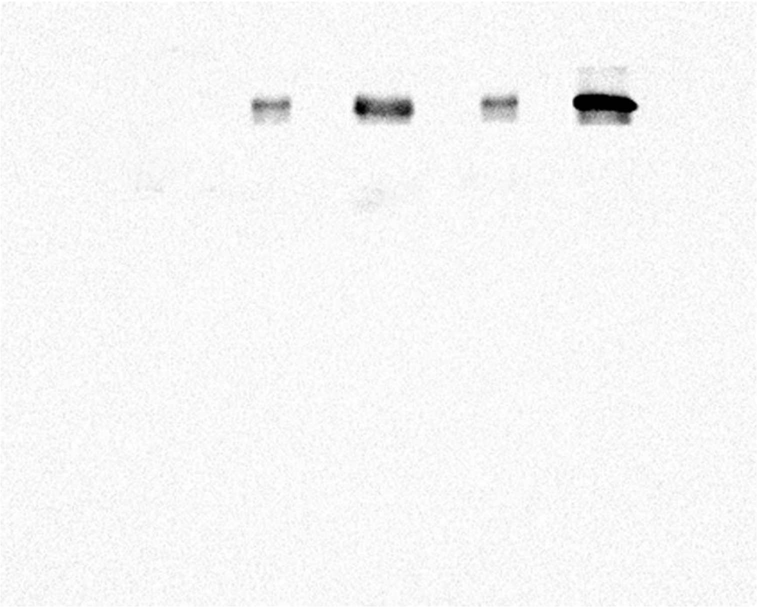

5A, B TAZ

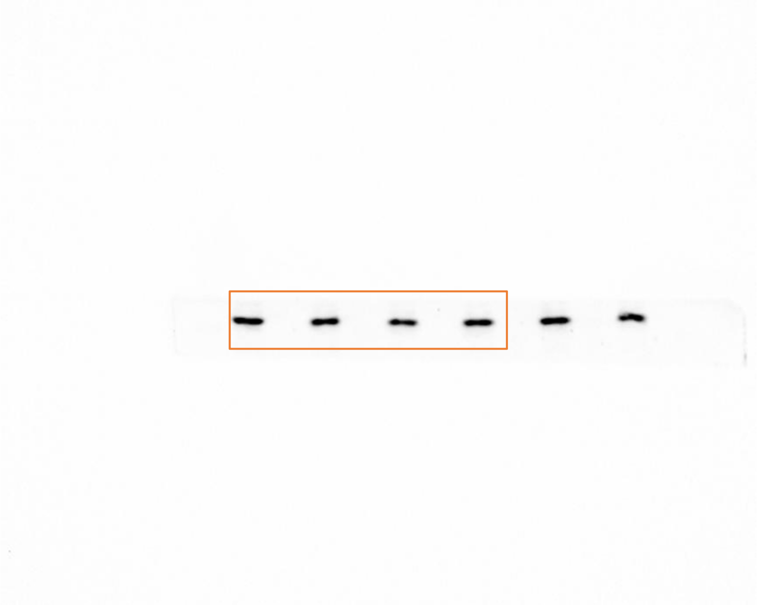

5A, B PSMA1

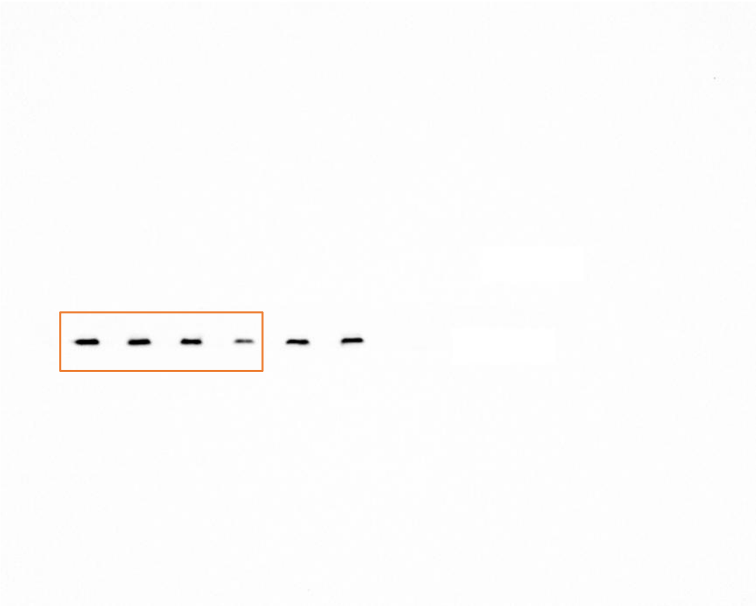

5B Flag

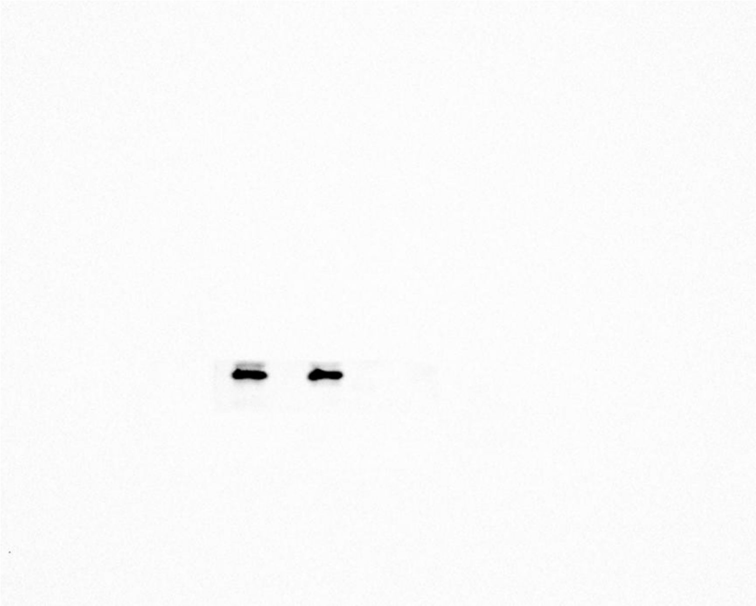

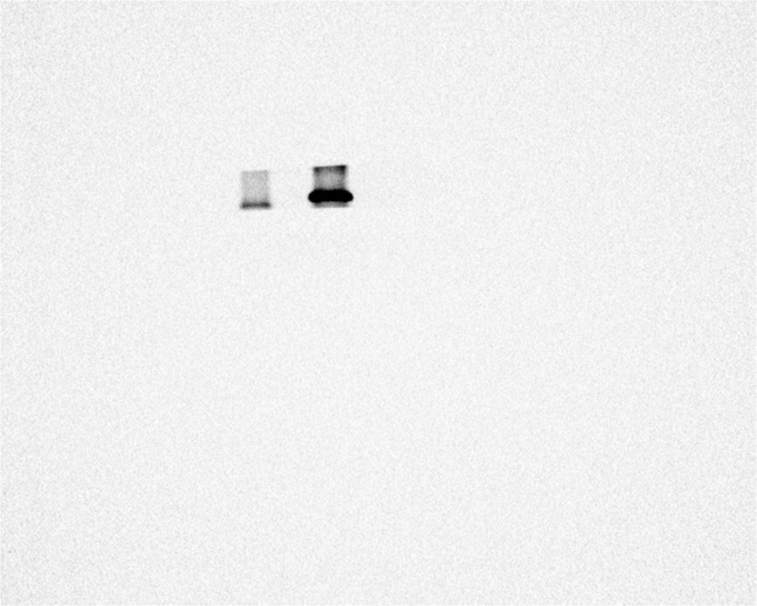

5B Myc

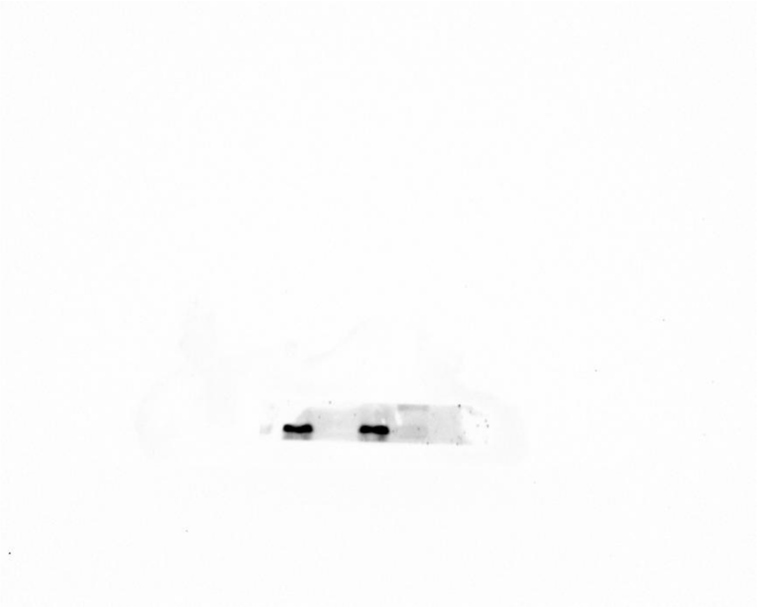

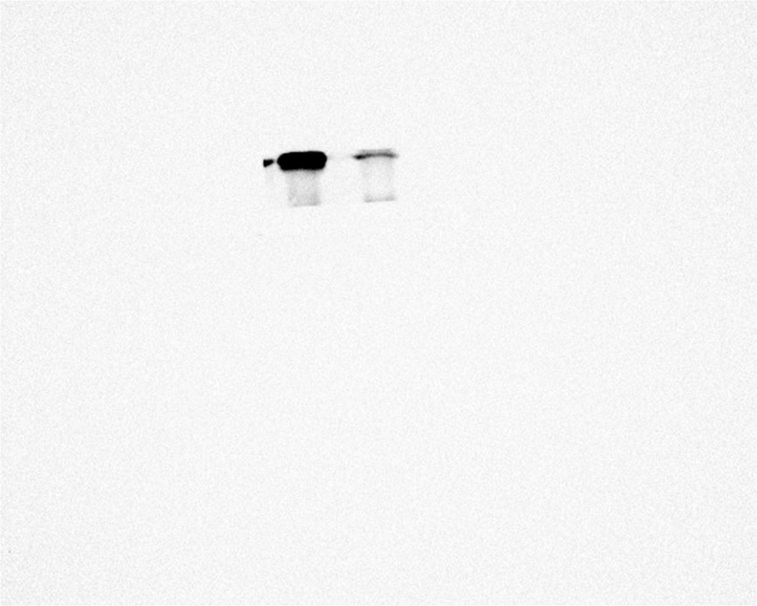

5E IP Flag

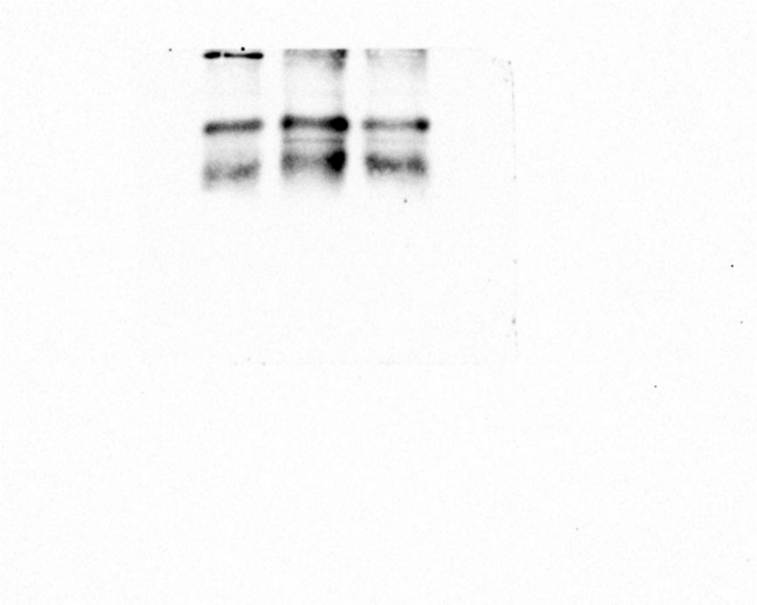

5E imput Flag

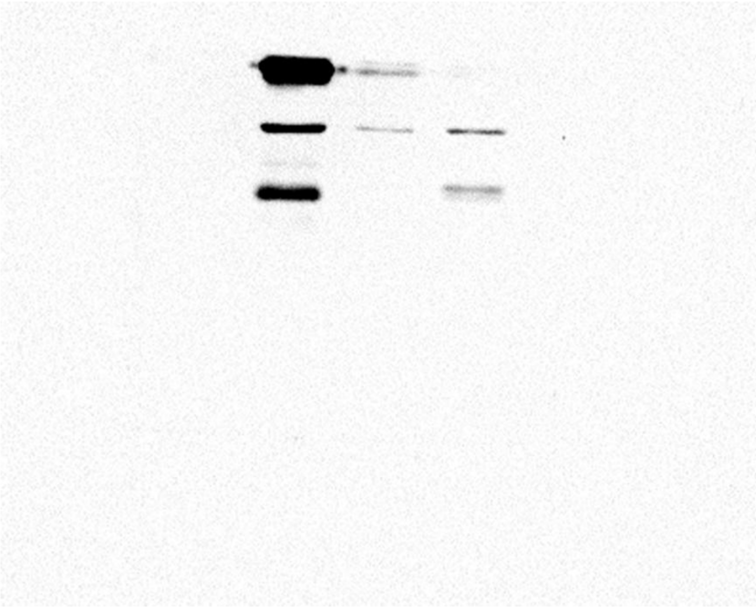

5E β-actin

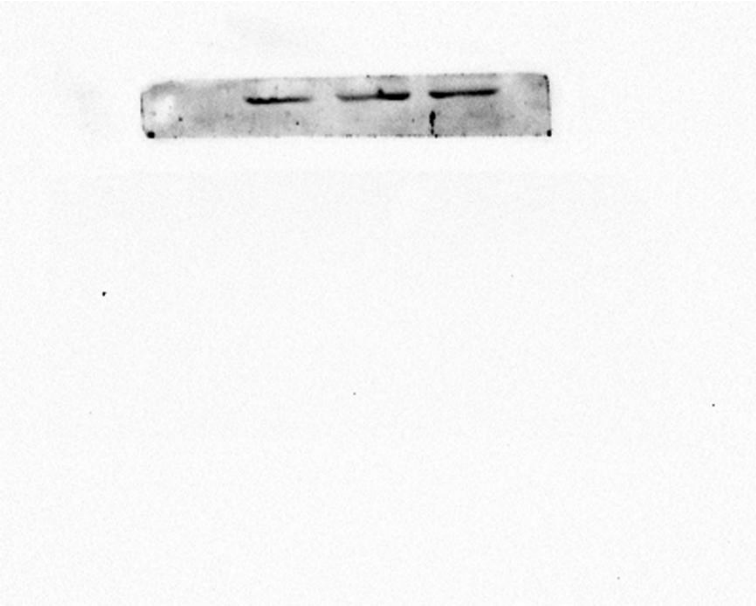

5E IP Myc

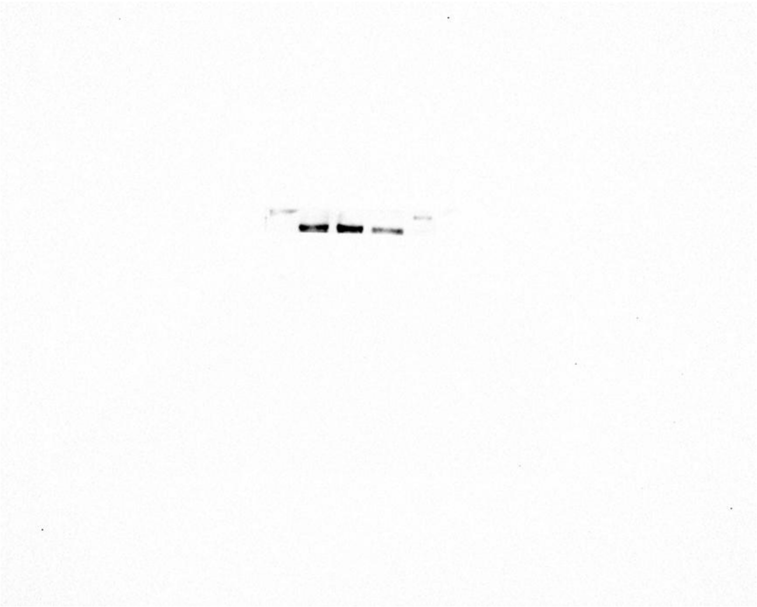

5E input Myc

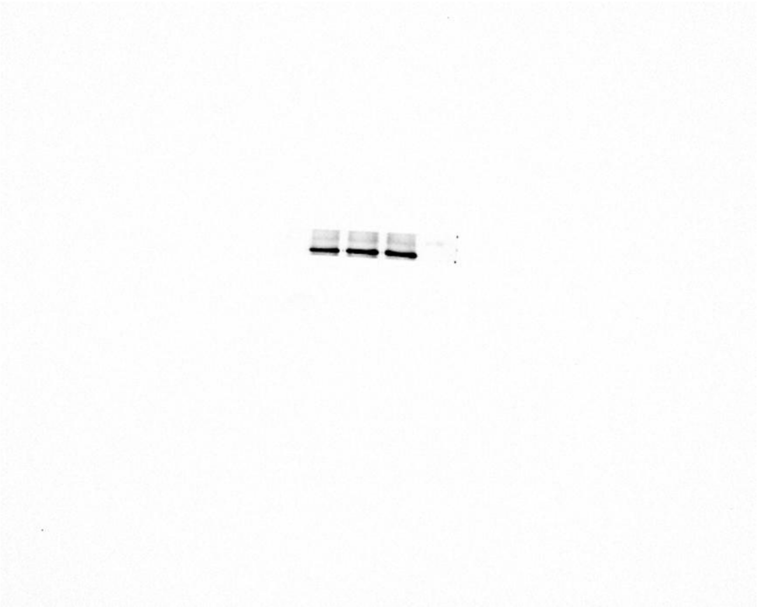

5E IP Myc

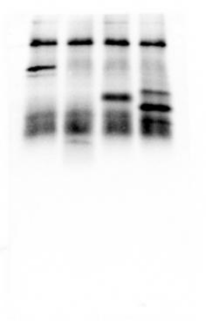

5E input Myc

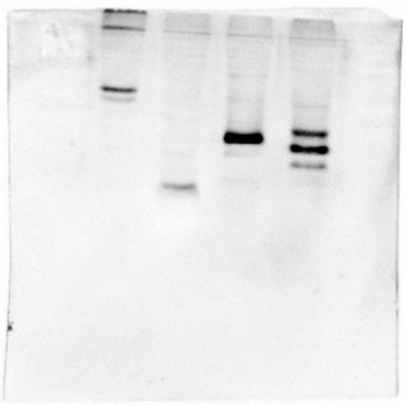

5E IP Flag

5E input Flag

5E β-actin

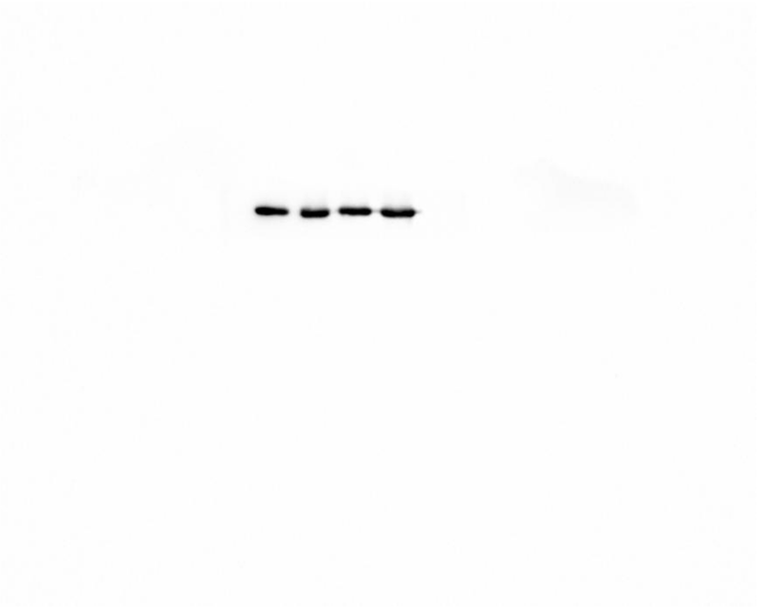

5F IP HA

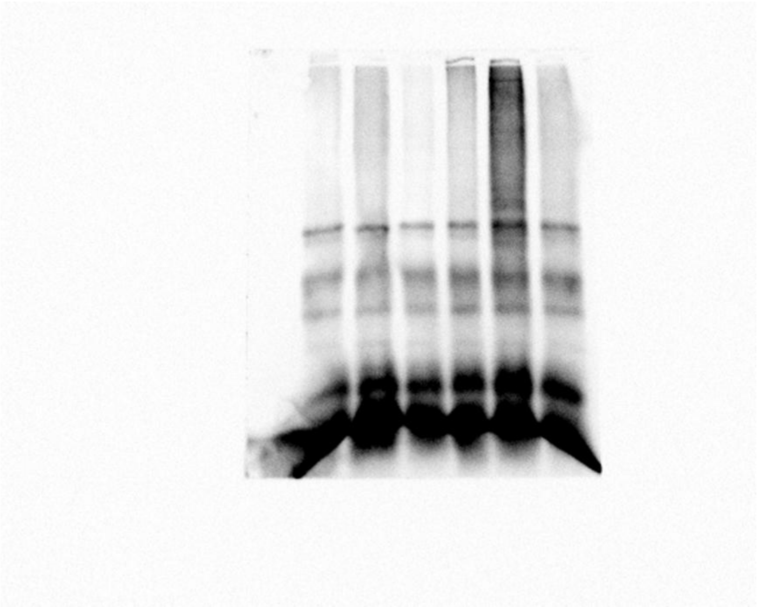

5F IP Myc

5F input Myc

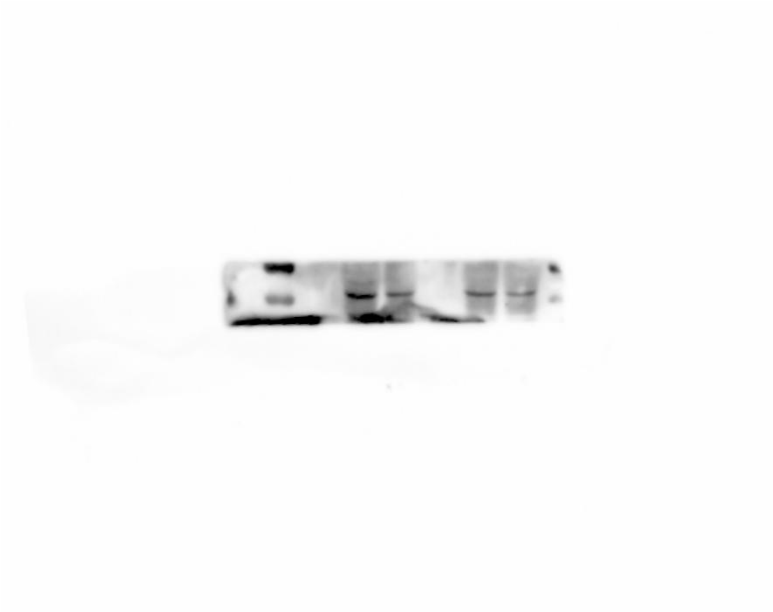

5F input Flag

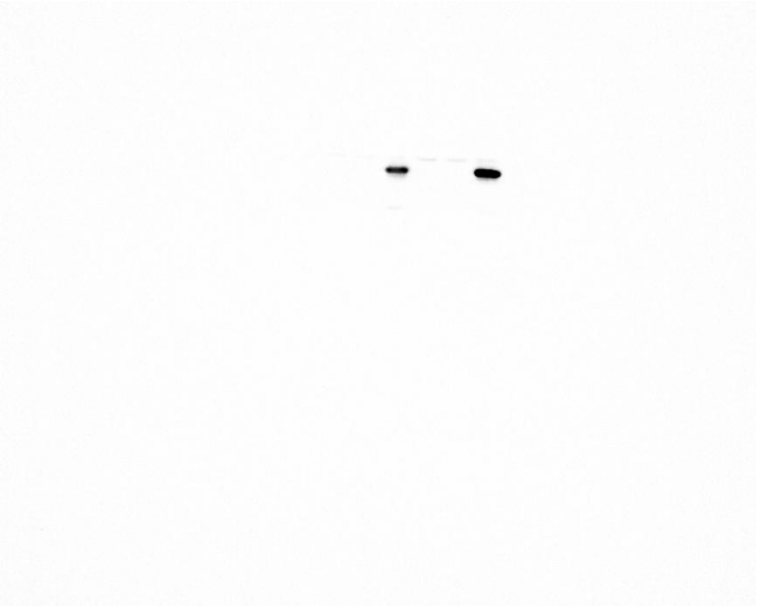

5F β-actin

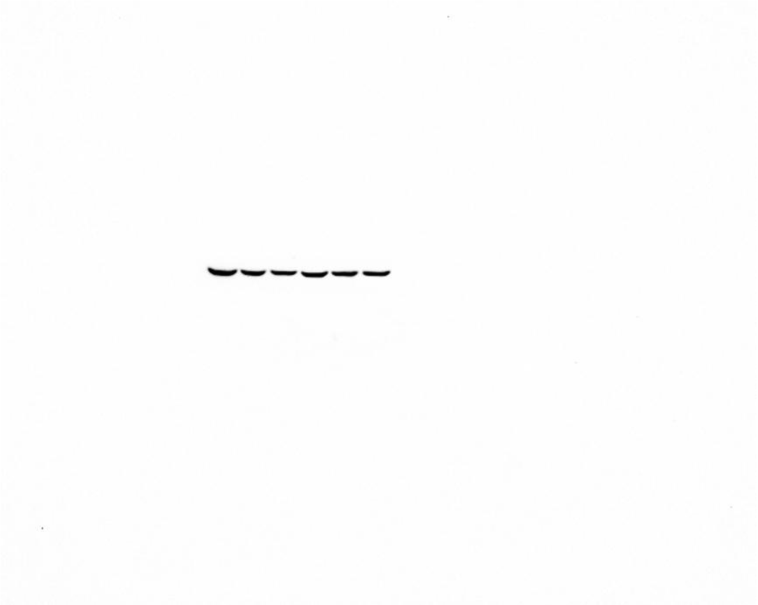

5G IP HA

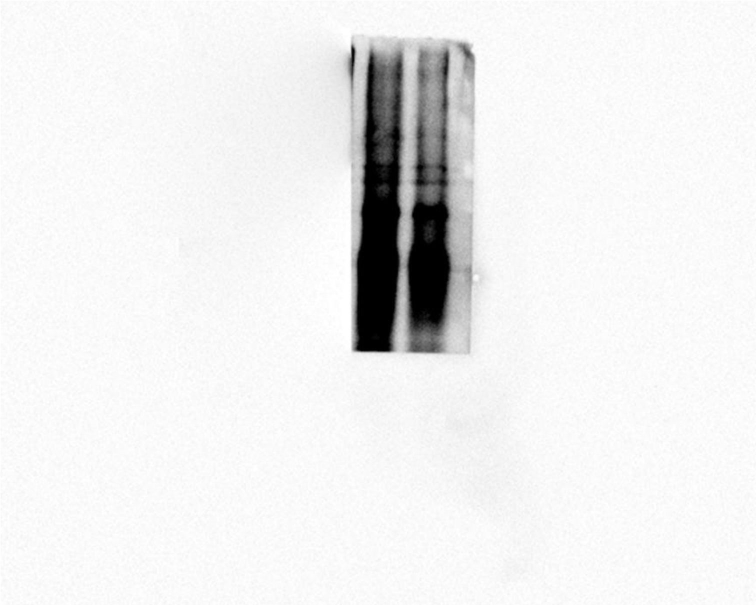

5G IP Myc

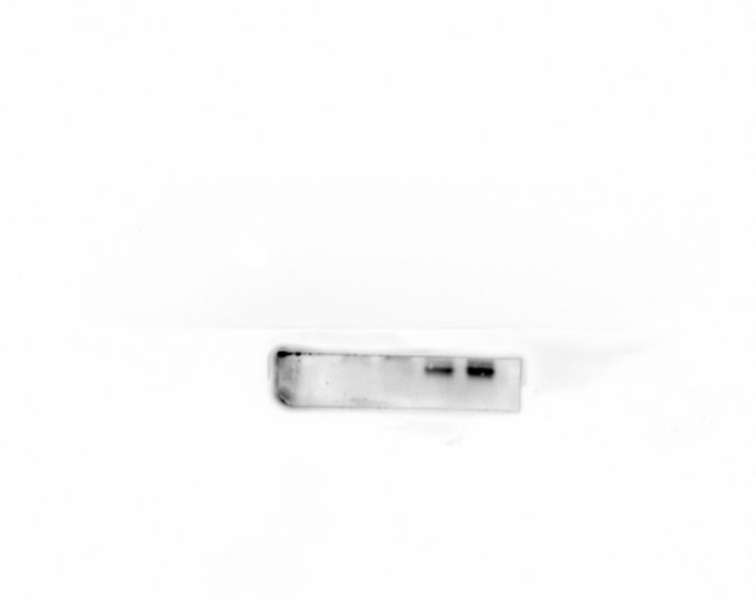

5G input Myc

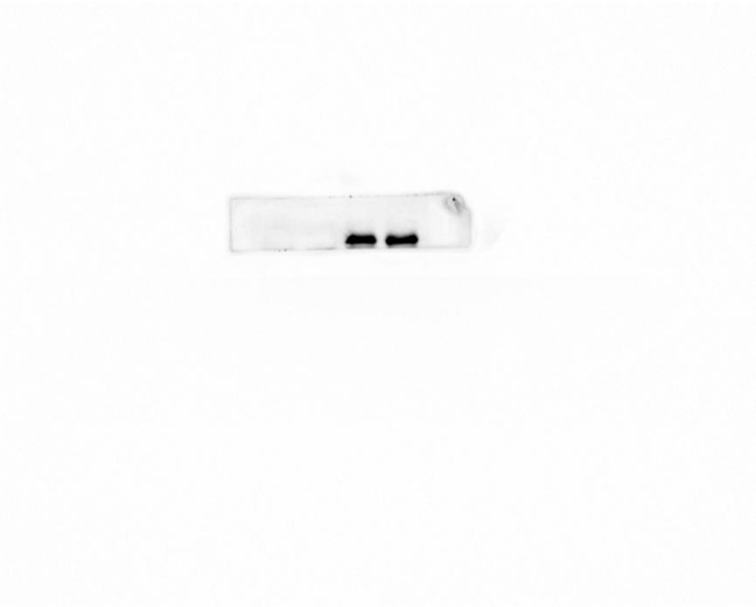

5G β-actin

5G input Flag

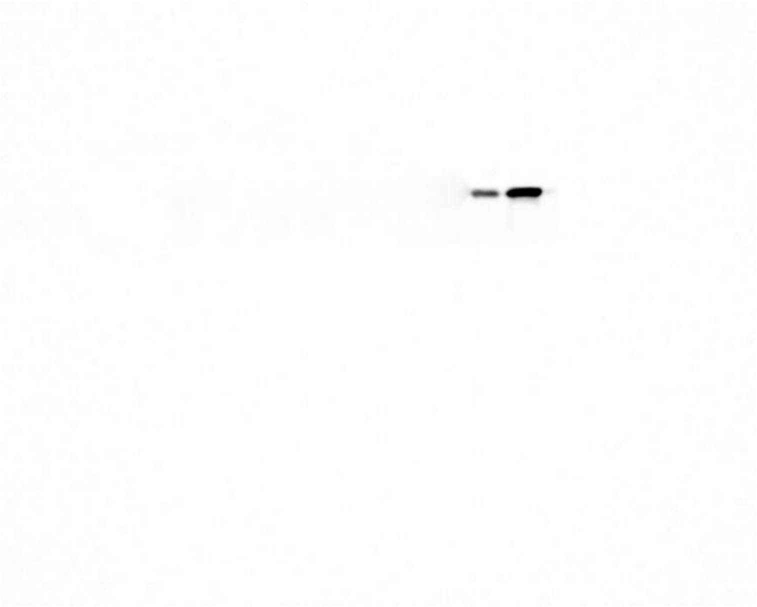

5H IP HA

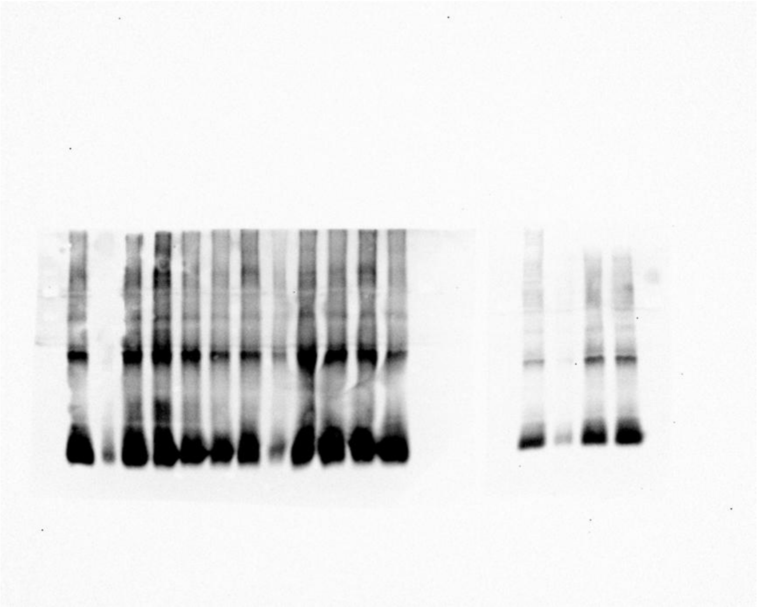

5H input Myc

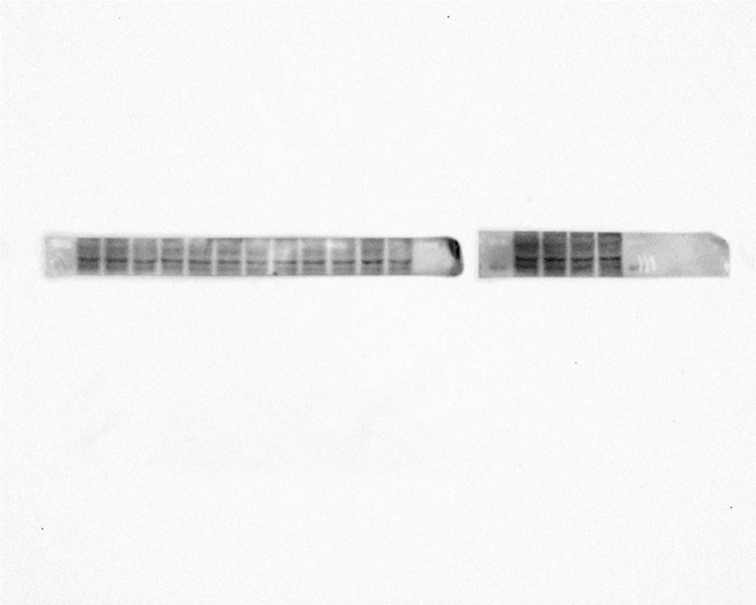

5H β-actin

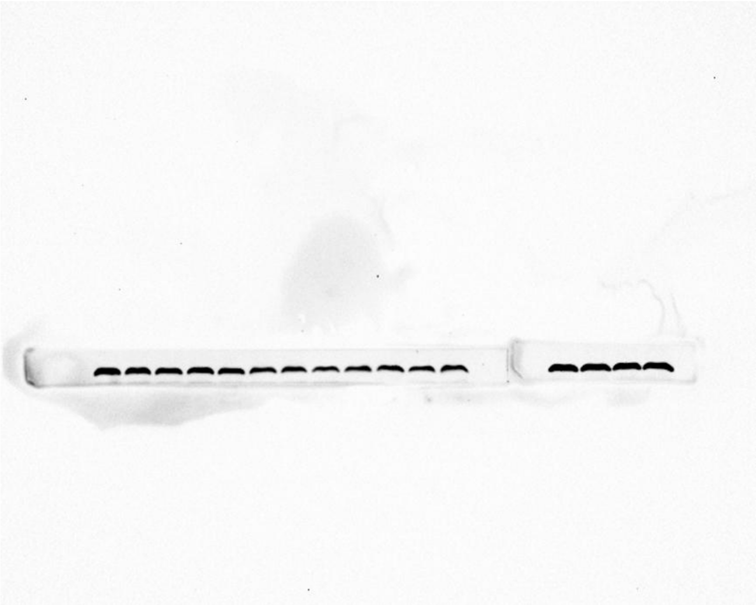

5H input Flag

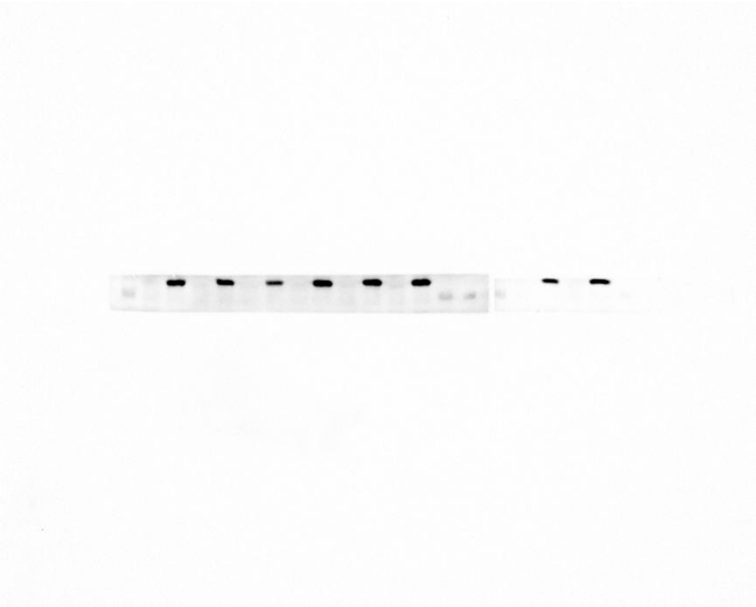

5H IP Myc

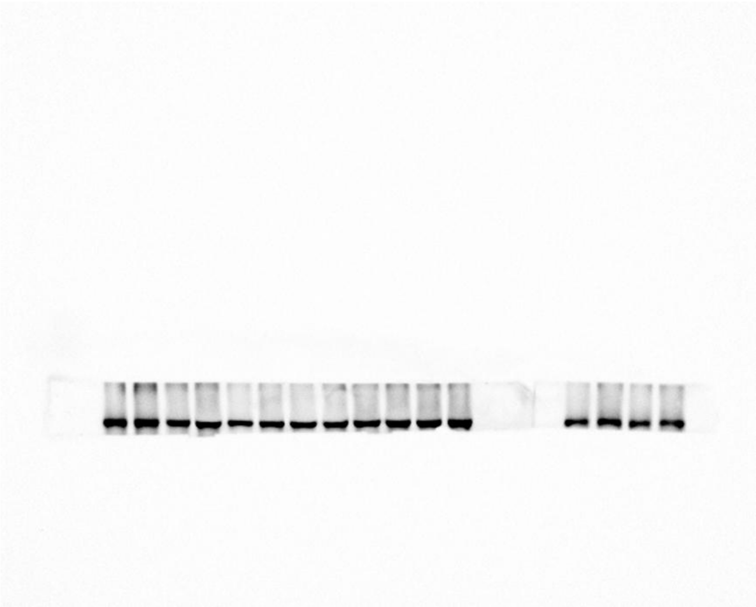

Figure 6

6E PCNA

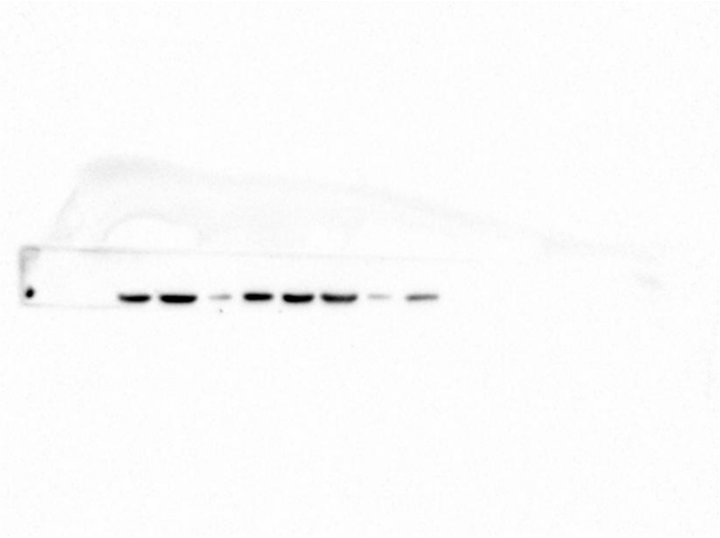

6E PSMA1

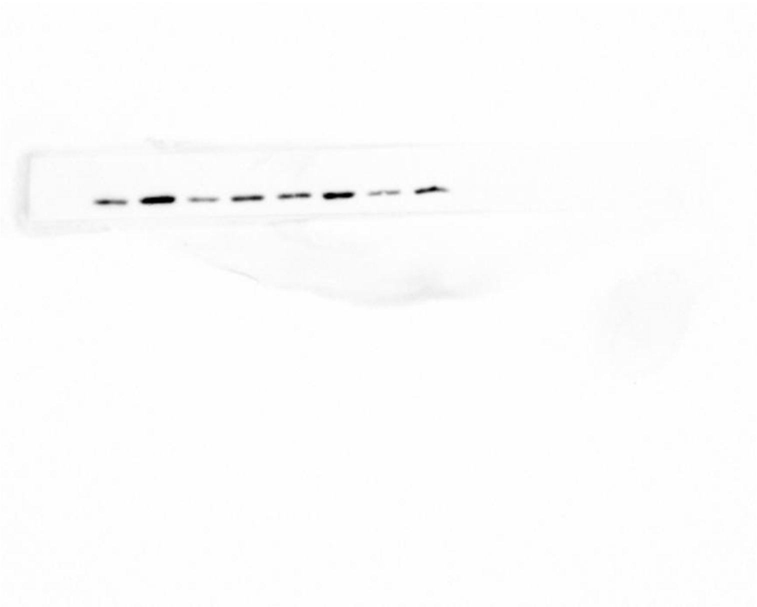

6E β-actin

6E C-Myc

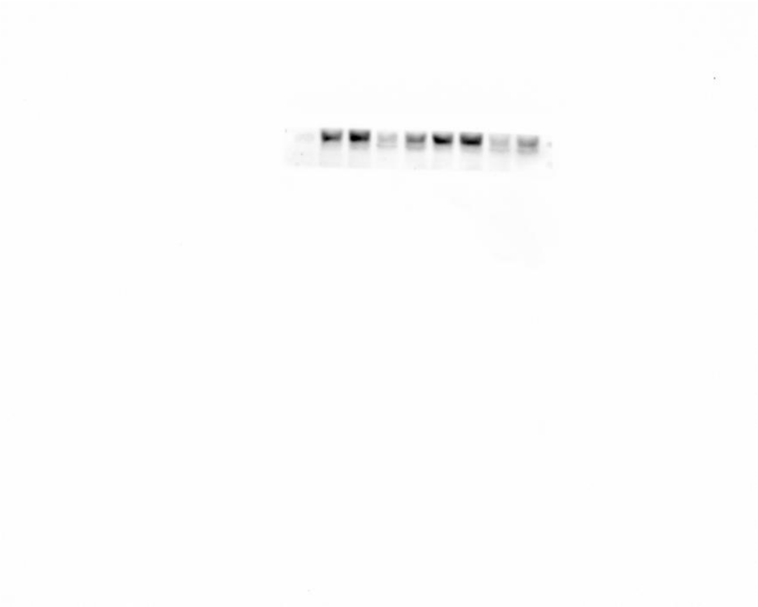

6E TAZ

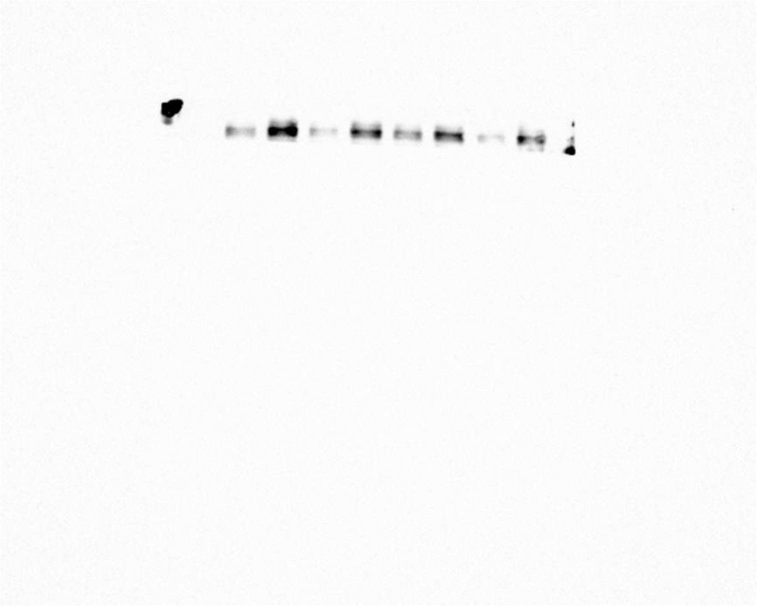

6E YAP

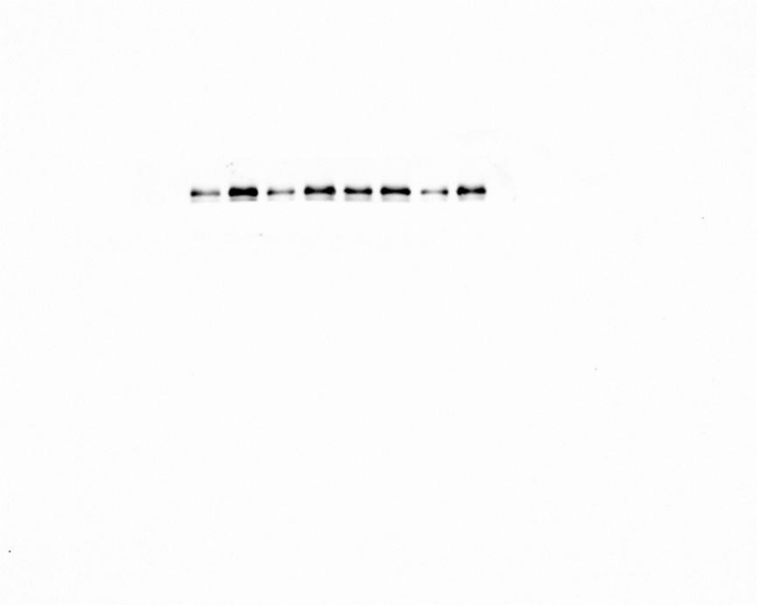

Figure 7

7H TAZ

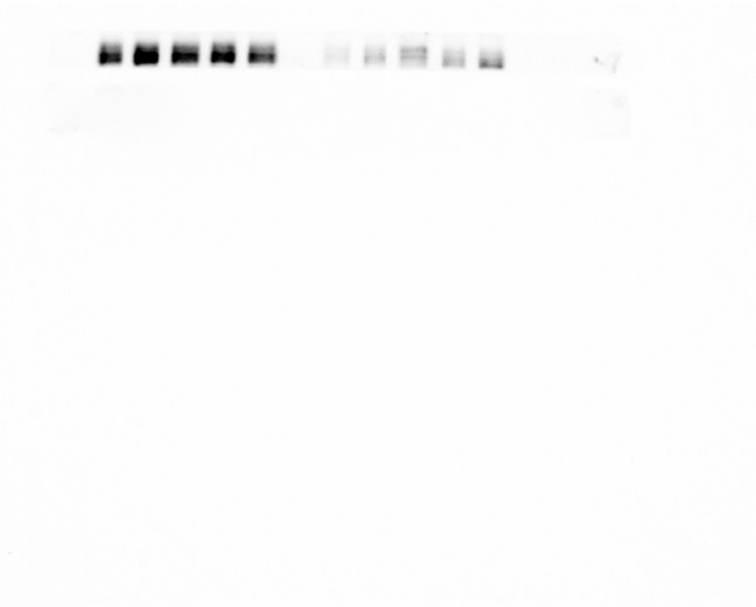

7H Ki67

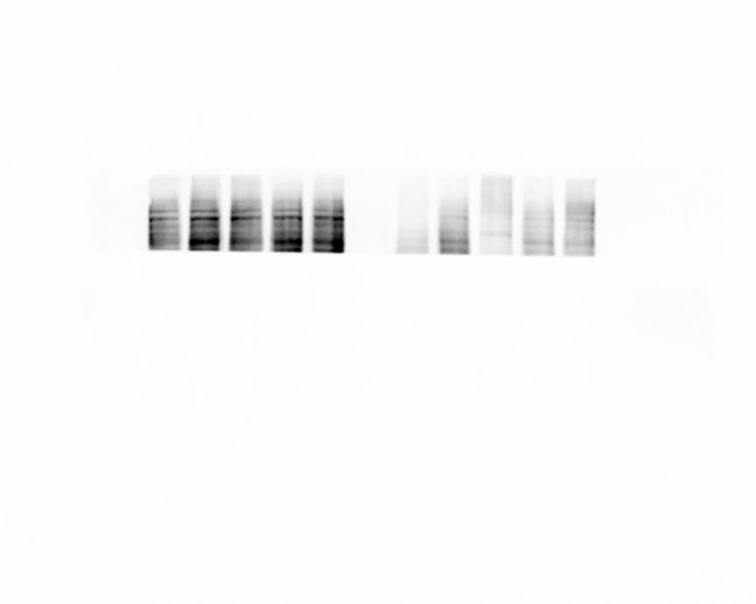

7H PSMA1

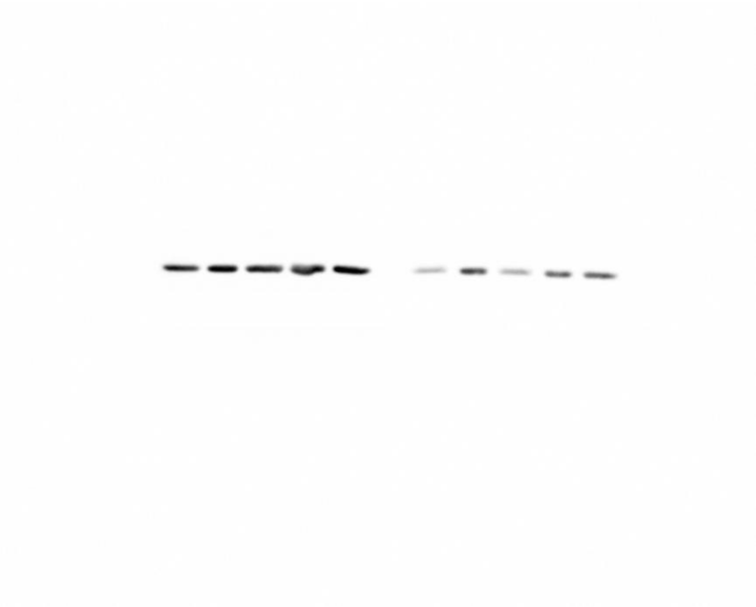

7H YAP

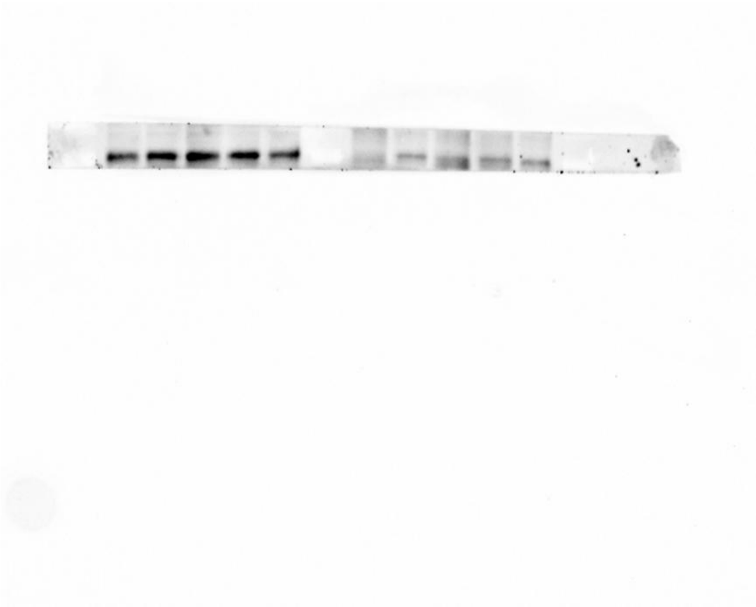

7H β-actin

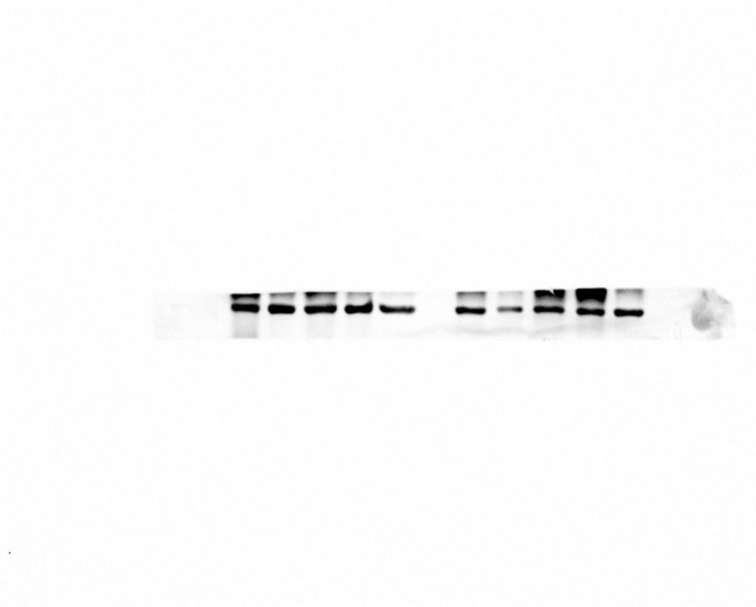

7H C-Myc

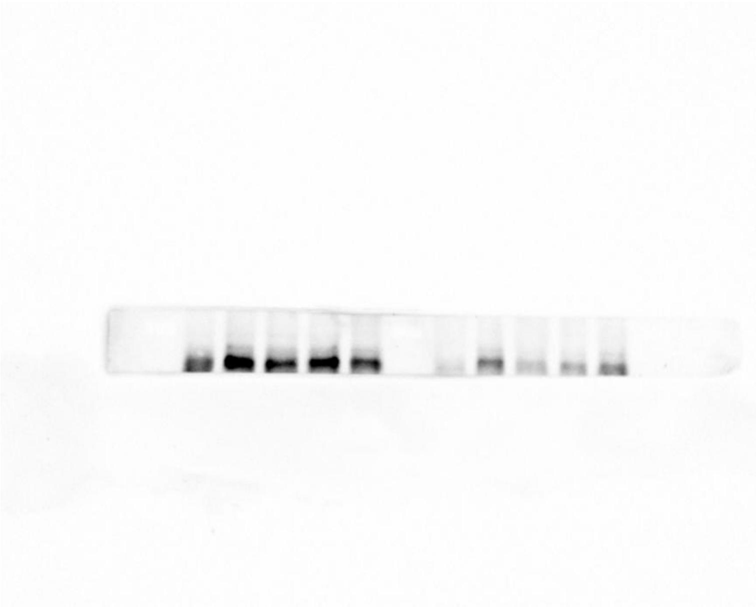

7H PCNA

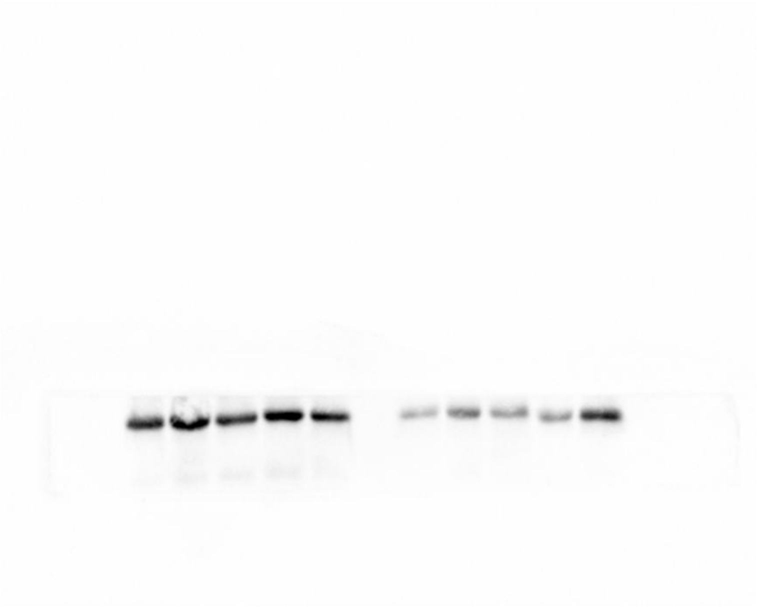

Supplement: Supplementary file 2 — Original Data File [file 41419_2022_5417_MOESM2_ESM.pdf]
