# Supplementary figures and images for: PSMA1 mediates tumor progression and poor prognosis of gastric carcinoma by deubiquitinating and stabilizing TAZ
Source: Cell Death Dis. 2022 Nov 23;13(11):989. doi: 10.1038/s41419-022-05417-0 (PMC9691733; doi:10.1038/s41419-022-05417-0)

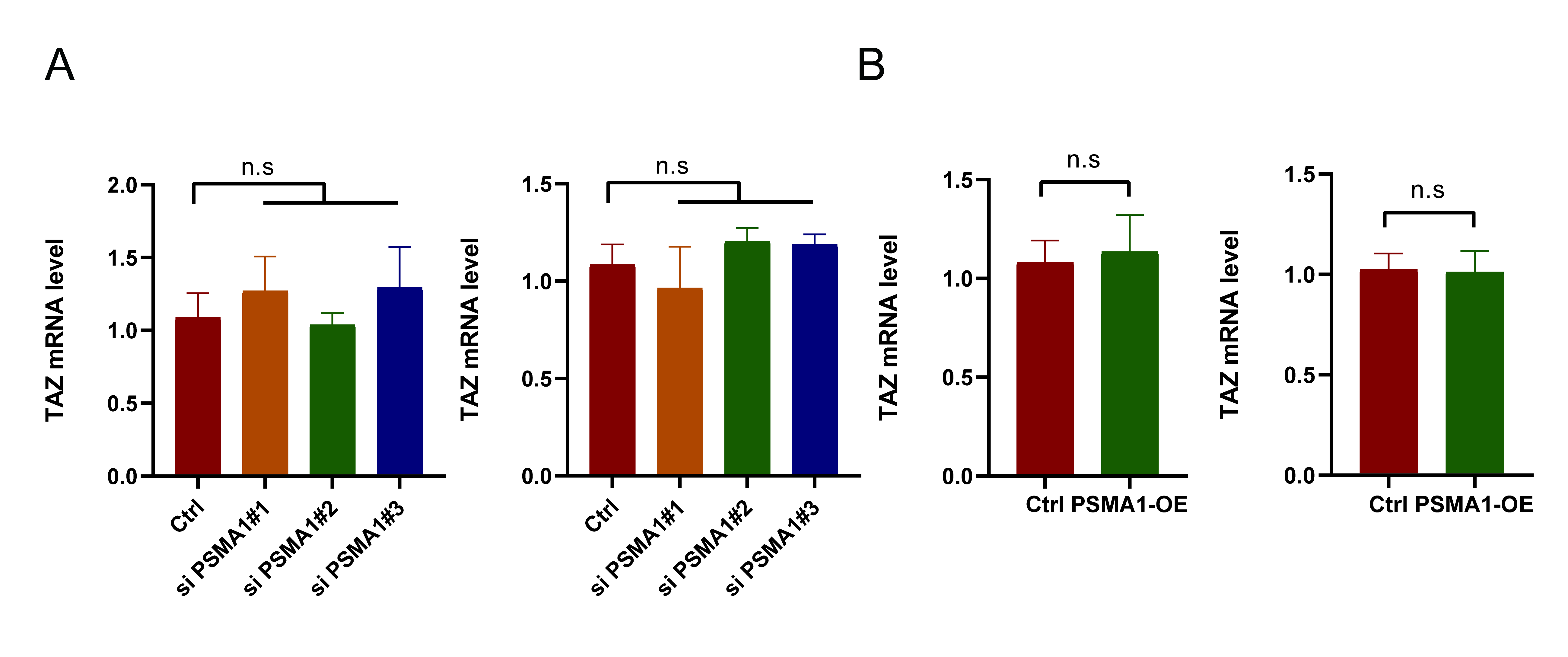

Supplement: Supplementary file 3 — Supplementary Figure 1 [file 41419_2022_5417_MOESM3_ESM.tif]

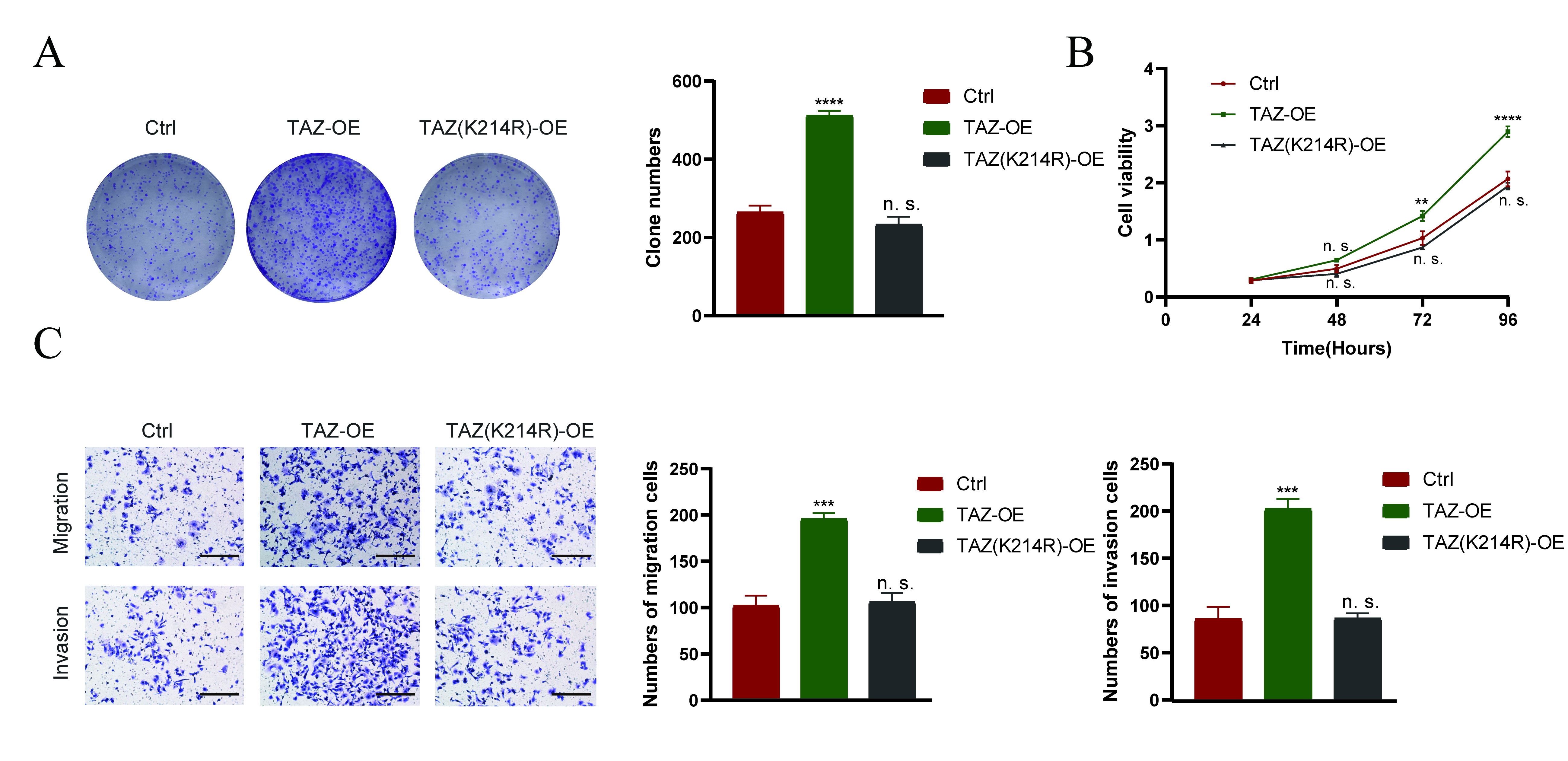

Supplement: Supplementary file 5 — Supplementary Figure 3 [file 41419_2022_5417_MOESM5_ESM.tif]

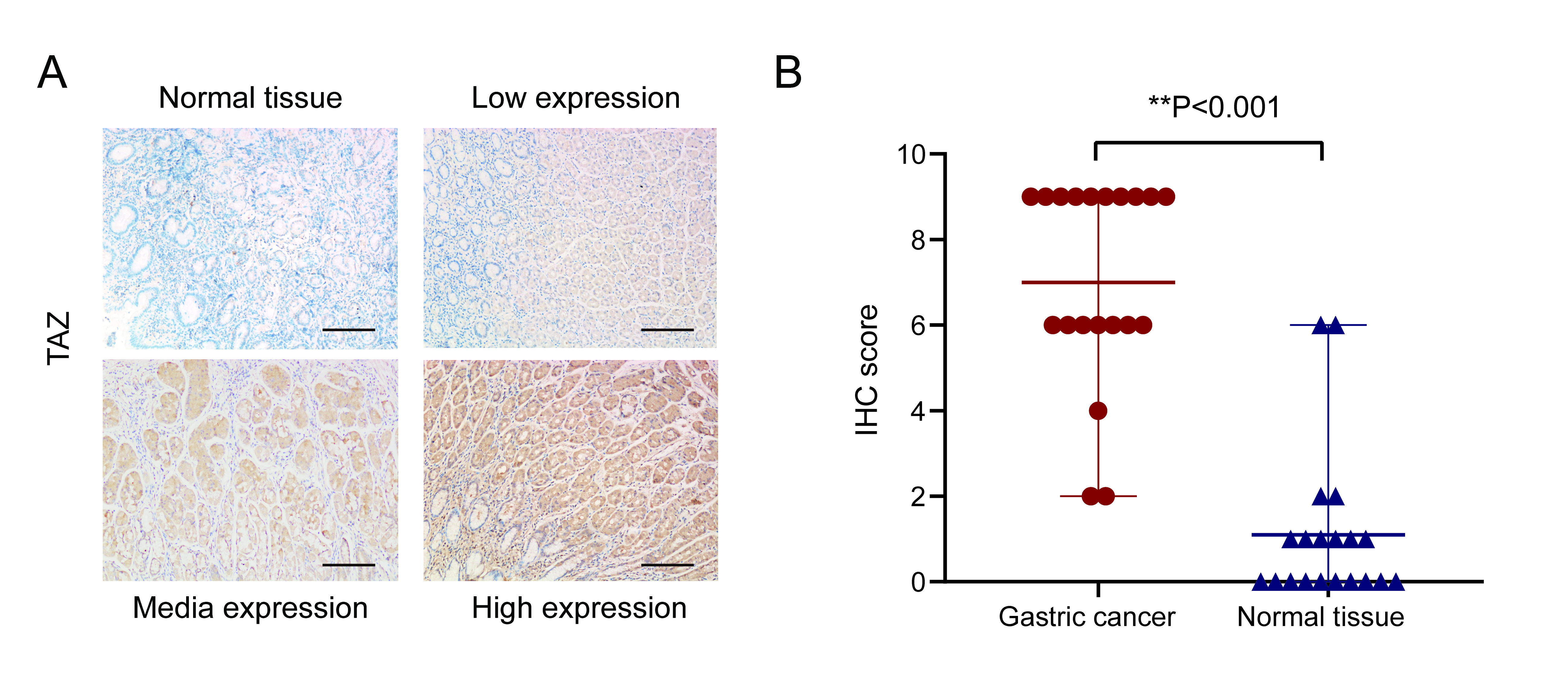

Supplement: Supplementary file 6 — Supplementary Figure 4 [file 41419_2022_5417_MOESM6_ESM.tif]
